# Macrophage-enriched *Sectm1a* promotes efficient efferocytosis to attenuate ischemia/reperfusion-induced cardiac injury

**DOI:** 10.1172/jci.insight.173832

**Published:** 2024-03-08

**Authors:** Xiaohong Wang, Wa Du, Yutian Li, Hui-Hui Yang, Yu Zhang, Rubab Akbar, Hannah Morgan, Tianqing Peng, Jing Chen, Sakthivel Sadayappan, Yueh-Chiang Hu, Yanbo Fan, Wei Huang, Guo-Chang Fan

**Affiliations:** 1 Department of Pharmacology and Systems Physiology,; 2 Department of Cancer Biology, and; 3 Division of Cardiovascular Health and Disease, Department of Internal Medicine, University of Cincinnati College of Medicine, Cincinnati, Ohio, USA.; 4 Centre for Critical Illness Research, Lawson Health Research Institute, London, Ontario, Canada.; 5 Division of Biomedical Informatics and; 6 Transgenic Animal and Genome Editing Facility, Division of Developmental Biology, Cincinnati Children’s Hospital Medical Center, Cincinnati, Ohio, USA.

**Keywords:** Cardiology, Immunology, Cardiovascular disease, Cellular immune response, Macrophages

## Abstract

Efficient clearance and degradation of apoptotic cardiomyocytes by macrophages (collectively termed efferocytosis) is critical for inflammation resolution and restoration of cardiac function after myocardial ischemia/reperfusion (I/R). Here, we define secreted and transmembrane protein 1a (*Sectm1a*), a cardiac macrophage–enriched gene, as a modulator of macrophage efferocytosis in I/R-injured hearts. Upon myocardial I/R, *Sectm1a*-KO mice exhibited impaired macrophage efferocytosis, leading to massive accumulation of apoptotic cardiomyocytes, cardiac inflammation, fibrosis, and consequently, exaggerated cardiac dysfunction. By contrast, therapeutic administration of recombinant SECTM1A protein significantly enhanced macrophage efferocytosis and improved cardiac function. Mechanistically, SECTM1A could elicit autocrine effects on the activation of glucocorticoid-induced TNF receptor (GITR) at the surface of macrophages, leading to the upregulation of liver X receptor α (*LXRα*) and its downstream efferocytosis-related genes and lysosomal enzyme genes. Our study suggests that *Sectm1a*-mediated activation of the *Gitr*/*LXRα* axis could be a promising approach to enhance macrophage efferocytosis for the treatment of myocardial I/R injury.

## Abstract 2

Sectm1a regulates macrophage efferocytosis in ischemic/reperfused hearts.

## Graphical abstract

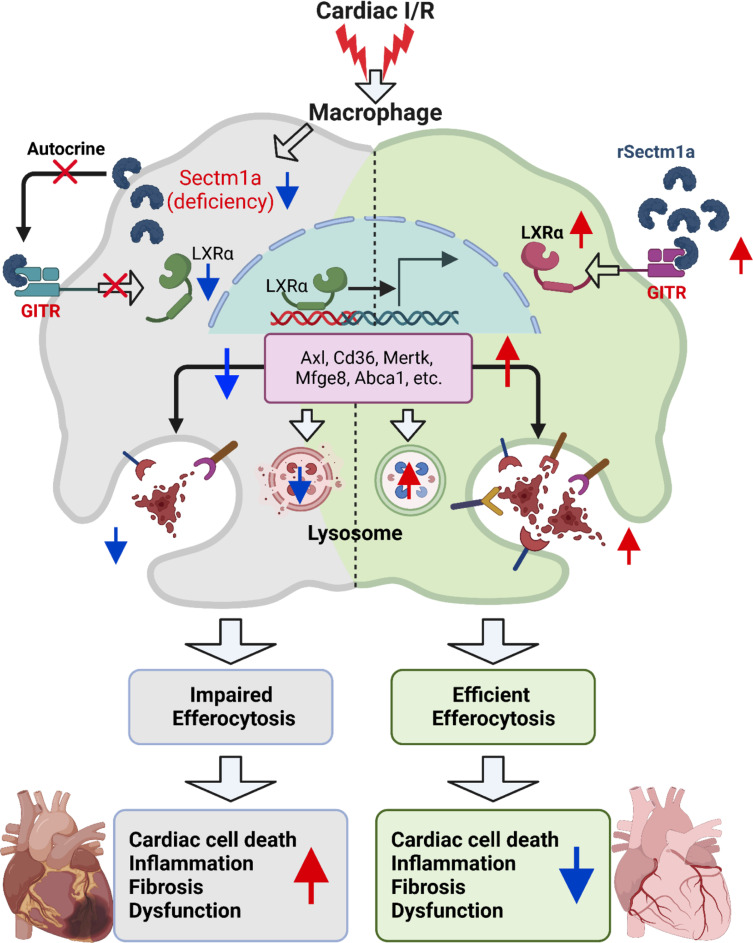

## Introduction

Acute myocardial infarction (MI) is a leading cause of death worldwide (1). Currently, it is well appreciated that timely reperfusion via thrombolytic agents or primary percutaneous coronary intervention represents the most effective remedy for patients with MI (2–4). However, the reperfusion of ischemic heart can trigger additional damage, such as extensive cardiomyocyte death and subsequent cardiac inflammation (5–7). Indeed, during the early phase of reperfusion, the increased amount of necrotic and apoptotic cardiomyocytes is a critical culprit for the progress of severe adverse remodeling and heart failure (7–9). Therefore, the efficient clearance of dead cardiomyocytes, termed efferocytosis, during ischemia/reperfusion (I/R) would be favorable for repairing myocardial I/R injury.

Efferocytosis is the processing and degradation of dead cells (i.e., apoptotic or necrotic) by either professional or nonprofessional phagocytes through phagocytic endocytosis (10–12). At present, it is well recognized that macrophages are the most common professional phagocytes that are capable of rapidly ingesting and processing multiple corpses in succession (10–12). In adult human and mouse hearts, macrophages that account for approximately 7% of the nonmyocyte population under healthy conditions are indispensable for the maintenance of heart function and homeostasis (13, 14). In the setting of cardiac I/R, cardiomyocyte death is rampant, leading to the high ratio of dying cells to phagocytes that demands rapid uptake and efficient clearance of the corpse-derived cellular materials (13, 14). However, accumulating evidence has indicated that the efferocytosis capacity of cardiac macrophages is impaired in the I/R hearts, which causes secondary damage and eventually heart failure (15–18). Such I/R-induced defective efferocytosis may be ascribed to, at least in part, the ability of dead cardiomyocytes to release a few proteinases (e.g., ADAM metallopeptidase domain 17), leading to the cleavage of MER proto-oncogene tyrosine kinase (Mertk), a macrophage receptor known to be essential for the clearance of dead adult cardiomyocytes following I/R (18–20). Along this line, several recent studies have identified multiple mediators (e.g., resolvin D1, *miR-26a*, and *miR-126*) to limit *Mertk* shedding from macrophages for enhancing efferocytosis (21–23). Nonetheless, the molecular mechanism underlying the defective efferocytosis observed in I/R hearts remains obscure. In addition, it would be very significant to explore any novel factors that promote efficient efferocytosis to repair damaged hearts after I/R.

Secreted and transmembrane protein 1 (SECTM1) was initially identified on human chromosome 17q25 as a Golgi-associated protein with transmembrane and secreted isoforms (24). The mouse genome contains 2 *Sectm1* genes, called *Sectm1a* and *Sectm1b*, with the greatest homology between mouse *Sectm1a* and human *SECTM1* (25). Both human SECTM1 and mouse SECTM1A have been implicated as an alternative CD7 ligand to costimulate T cell proliferation (25, 26). Interestingly, mouse *Sectm1a* has also been speculated to interact with glucocorticoid-induced TNF receptor (GITR) in T cells and enhance their proliferation (25). Recently, we found that *Sectm1a* plays an essential role in macrophage phagocytosis to eradicate bacteria from septic mice (27) and facilitates protection against sepsis-induced organ damage (28). Furthermore, we observed that deficiency of *Sectm1a* promotes inflammatory macrophage phenotype and consequently aggravates inflammation-triggered cardiac dysfunction through disruption of liver X receptor α (*LXRα*) signaling in macrophages (29). However, whether *Sectm1a* affects macrophage efferocytosis to clear dead cells from I/R hearts has never been investigated.

In this study, we first analyzed a human cardiac single-nucleus RNA-sequencing (RNA-Seq) database and mouse cardiac single-cell RNA-Seq database and found that both human *SECTM1* and mouse *Sectm1a* are highly enriched in cardiac macrophages. Next, using in vitro and in vivo approaches, we demonstrated that *Sectm1a* is required for macrophage efferocytosis to effectively clear dead cells from I/R hearts. Mechanistically, we defined a link of *Sectm1a*/*Gitr* to the *LXRα* signaling in controlling macrophage efferocytosis. Last, we tested the therapeutic effects of recombinant SECTM1A protein (rSec) in I/R hearts. Our study presented here provides the evidence to show *Sectm1a* as a regulator of macrophage efferocytosis and an important factor for the treatment of myocardial I/R injury.

## Results

### The expression of Sectm1a, a cardiac macrophage–enriched gene, is dynamically altered in macrophages upon myocardial I/R and treatment with dead cells.

**Figure 1:**
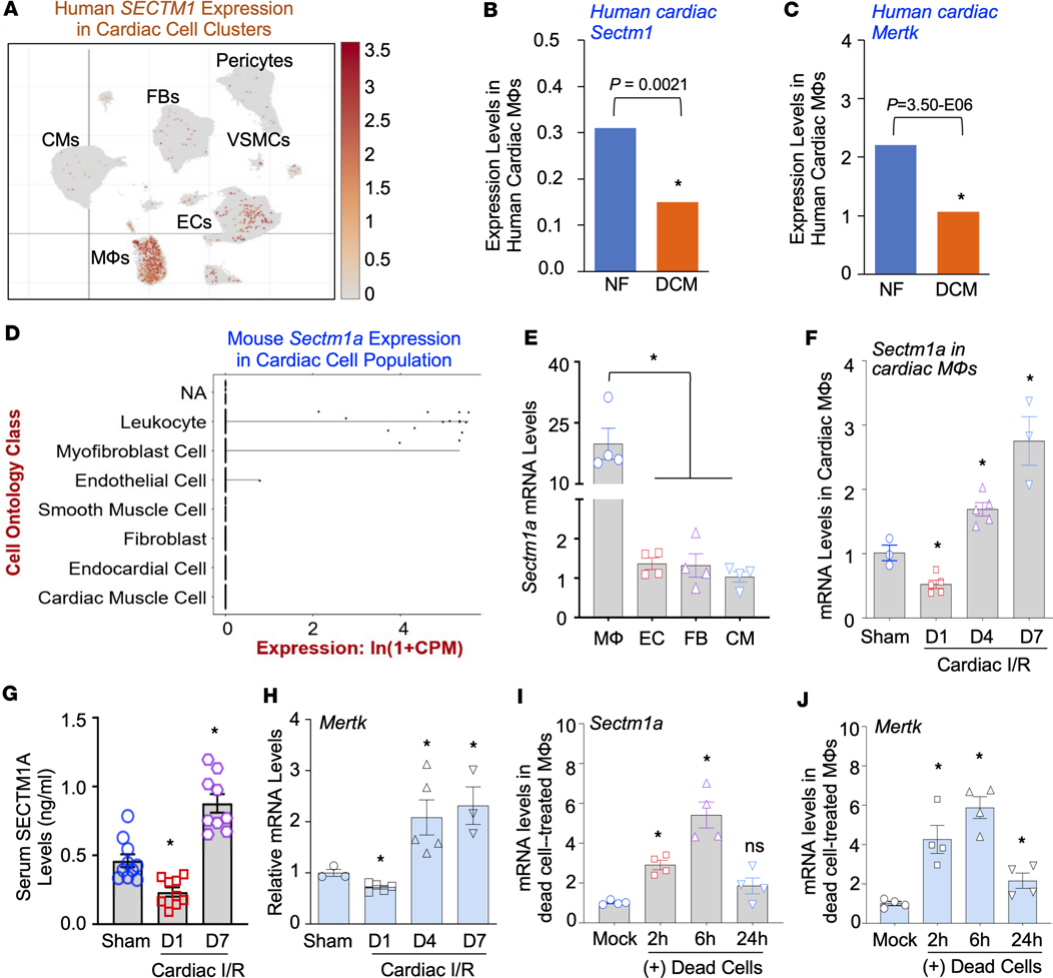
The dynamic expression of Sectm1/a in cardiac macrophages upon myocardial I/R or challenge with dying/dead cells. (**A**) Single-nucleus transcriptome data reveal human cardiac clusters of SECTM1 expression (*n* = 185,441 nuclei collected from 16 hearts). (**B** and **C**) Average mRNA levels of SECTM1 (**B**) and MERTK (**C**) in cardiac macrophages collected from healthy nonfailure (NF) and patients with dilated cardiomyopathy (DCM), analyzed by the above single-nucleus RNA-Seq data (34). (*n* =171,996 nuclei for DCM group, *n* = 185,411 nuclei for healthy NF group; *, *P* < 0.05.) (**D**) Single-cell RNA-Seq data from the Tabula Muris project (35) with t-distributed stochastic neighbor embedding (t-SNE) plot of all cells isolated from murine hearts by FACS show that Sectm1a is highly enriched in cardiac leukocyte cluster. (**E**) RT-qPCR analysis of Sectm1a mRNA levels in macrophages, ECs (endothelial cells), FBs (fibroblasts), and CMs (cardiomyocytes) isolated from murine hearts (*n* = 4; *, *P* < 0.05). (**F**) The expression levels of Sectm1a were determined in cardiac macrophages isolated from mice at the time points of day 1 (D1), day 4 (D4), and day 7 (D7) after myocardial I/R (*n* = 3–5; *, *P* < 0.05 vs. sham). (**G**) Serum levels of SECTM1A were measured in I/R mice (*n* = 9–10; *, *P* < 0.05 vs. sham). (**H**) The expression levels of Mertk were measured in cardiac macrophages isolated from I/R mice (*n* = 3–5; *, *P* < 0.05 vs. sham). (**I** and **J**) The expression levels of (**I**) Sectm1a and (**J**) Mertk were measured in cultured mouse BMDMs at the indicated time points after incubation with dying/dead H9c2 cells (*n* = 4; *, *P* < 0.05 vs. mock). The expression of GAPDH was used as the internal control for RT-qPCR. All results are presented as mean ± SEM and analyzed by Student’s *t* test (**B** and **C**) or 1-way ANOVA (**E**–**J**). CPM, counts per million; RT-qPCR, reverse transcription quantitative PCR; VSMCs, vascular smooth muscle cells.

Both human *SECTM1* and mouse *Sectm1a* are ubiquitously expressed but with different tissue distributions, having the highest expression in the spleen (24, 25, 29). Further studies indicate that *Sectm1/a* is highly expressed in epithelial cells and leukocytes of the myeloid lineage but not detectable in lymphocytes (e.g., T and B cells, megakaryocytes) (30–32). Recent single-cell/nucleus RNA-Seq analysis reveals that the myocardium is composed of multiple cell types, including endothelial cells (ECs, ~29%), fibroblasts (FBs, ~25%), cardiomyocytes (CMs, ~23%), leukocytes/macrophages (~10%), neuronal cells (~7%), and others (smooth muscle cells, adipocytes, T cells, and B cells) (33). Therefore, to investigate the exact role of *Sectm1/a* in cardiac macrophage efferocytosis to clear dead cells from I/R heart, we first interrogated the transcript levels of *Sectm1/a* in cardiac cells within both human and mouse hearts by analyzing single-nucleus RNA-Seq profiles of human hearts (https://singlecell.broadinstitute.org/) (34) and single-cell transcriptomics of mouse hearts (Tabula Muris, https://tabula-muris.ds.czbiohub.org/) (35). Impressively, we found that human *SECTM1* was the highest enriched in macrophages among all cardiac cell types (Figure 1A), and the average expression levels of *SECTM1* were significantly reduced by 0.82-fold in cardiac macrophages only within human dilated cardiomyopathy (DCM), compared with healthy nonfailure (NF) hearts (Figure 1B). However, we checked the single-nucleus RNA-Seq database generated from human patients with hypertrophic cardiomyopathy and healthy donors, which revealed no changes of the SECTM1 mRNA levels in cardiac macrophages between 2 groups (see supplemental data from ref. 34). More interestingly, the average expression levels of *MERTK*, a cell surface receptor well known to be a marker of macrophage efferocytosis (22, 36–38), were coincidentally decreased by 1.92-fold in cardiac macrophages of human DCM, when compared with NF hearts (*P* < 0.001, Figure 1C). These data suggest that reduced *SECTM1* in cardiac macrophages may impair efferocytosis to remove dead cells from hearts. Like human *SECTM1* in cardiac macrophages, mouse *Sectm1a* was the highest expressed in cardiac leukocytes (Figure 1D), whereas *Sectm1b* was the most enriched in cardiac ECs (Supplemental Figure 1, A–C; supplemental material available online with this article; https://doi.org/10.1172/jci.insight.173832DS1), compared with other cardiac cell types. Next, we isolated ECs, FBs, CMs, and macrophages from mouse hearts to measure *Sectm1a* expression levels. Our RT-qPCR results clearly validated that the mRNA levels of *Sectm1a* in cardiac macrophages were approximately 20-fold higher, in comparison with the other 3 cardiac cell types (Figure 1E). Taken together, these data indicate that both human *SECTM1* and mouse *Sectm1a* are highly expressed in cardiac macrophages, implying their potential role in the macrophage-mediated clearance of dead cells from hearts upon stress/disease conditions.

Given that *Sectm1a* is highly enriched in cardiac macrophages, we next determined whether its expression is altered in cardiac macrophages after I/R. To this end, we isolated total macrophages from wild-type (WT) mouse hearts at day 1 (D1), day 4 (D4), and day 7 (D7) after I/R and extracted total macrophage RNAs for RT-qPCR. Cardiac macrophages isolated from sham-operated mice were used as controls. Please note here that we did not measure and compare Sectm1a levels between cardiac resident versus infiltrating macrophages within I/R hearts, as it has been reported that cardiac resident macrophages are initially lost at D1 post-MI, to be replaced by the infiltrating neutrophils and monocytes/macrophages (14). RT-qPCR results showed that the mRNA levels of *Sectm1a* were significantly decreased by 0.53-fold in cardiac macrophages at D1 post-I/R yet greatly increased by 1.69-fold at D4 and remarkably elevated by 2.75-fold at D7 post-I/R, compared with sham controls (Figure 1F). Consistently, using an ELISA kit, we observed that the serum levels of Sectm1a were significantly lower in mice at D1 post-I/R yet remarkably higher in mice at D7 post-I/R, compared with sham-operated mice (Figure 1G). In addition, we assessed the expression levels of *Mertk* in these cardiac macrophages (Figure 1H), which exhibited a similar alteration pattern as *Sectm1a* did. To further evaluate the expression levels of *Sectm1a* in macrophages challenged with dead cells, we cultured mouse bone marrow–derived macrophages (BMDMs), followed by incubation with dying/dead H9c2 cells for 2 hours, 6 hours, or 24 hours. Subsequently, the nonengulfed dying/dead cells were washed away, and BMDMs were collected at the indicated time points for the isolation of total RNAs. The dying/dead cells were prepared by treatment with H_2_O_2_ (1 mM, 2 hours) and validated by flow cytometry analysis with annexin V staining (Supplemental Figure 2, A and B). Our RT-qPCR results showed that mRNA levels of *Sectm1a* were significantly increased at 2 hours and peaked at 6 hours after dying/dead cell treatment, compared with mock-treated controls (Figure 1I). Likewise, the Sectm1a levels were much higher in the culture supernatants collected from BMDMs at 6-hour exposure to dead cells, compared with mock-treated supernatants (Supplemental Figure 2C). Of interest, a similar alteration pattern was observed in the expression levels of *Mertk* in BMDMs upon incubation with dying/dead cells (Figure 1J). Collectively, based on these data above, we hypothesized that *Sectm1a* may positively regulate macrophage efferocytosis to repair the damaged heart during I/R.

### Sectm1a deficiency impairs macrophage efferocytosis in vitro and in vivo.

**Figure 2:**
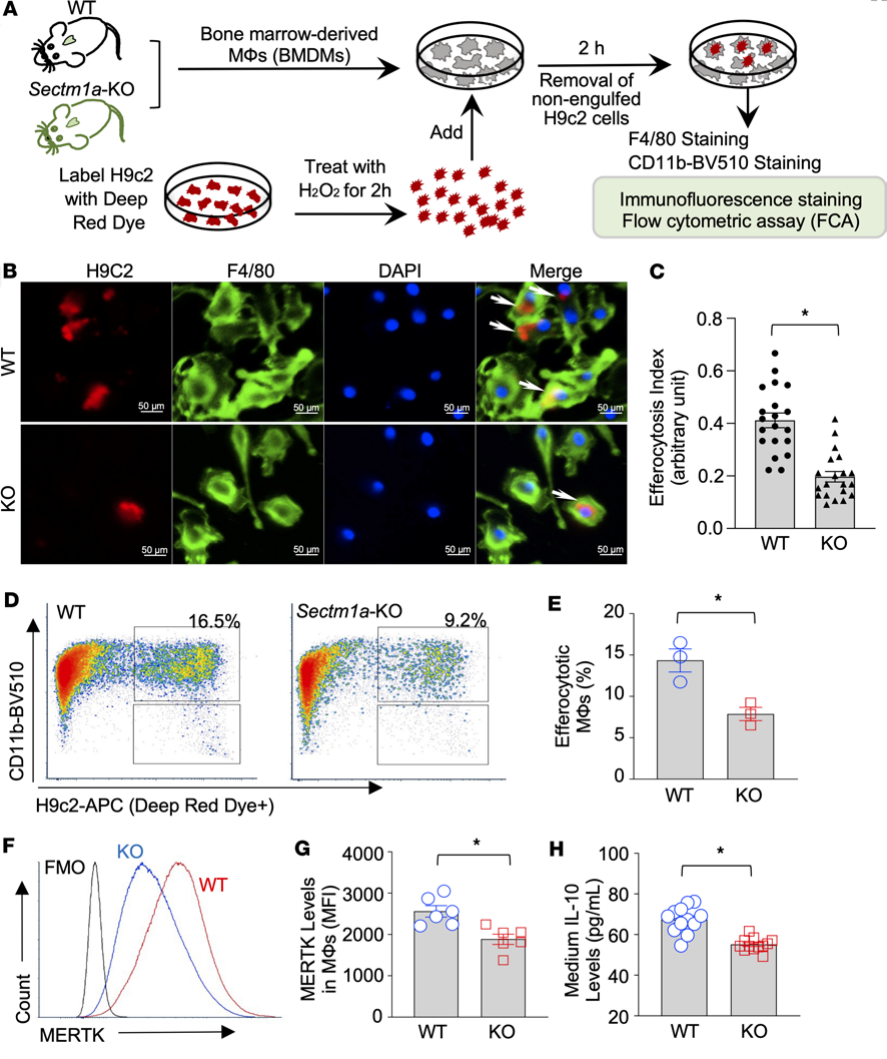
*Sectm1a* deficiency impairs macrophage efferocytosis in vitro. (**A**) Scheme of the in vitro efferocytosis model. BMDMs collected from WT and *Sectm1a*-knockout (KO) mice were exposed to red dye–labeled dead H9c2 cells for 2 hours, followed by removal of nonphagocytosed cells and subsequent fixation/staining with F4/80 or BV510-conjugated CD11b antibody for immunostaining and flow cytometry analysis, respectively. (**B**) Representative immunostaining and (**C**) their quantification results showing the reduced uptake of red dye–labeled dying/dead cells in KO macrophages, compared with WT (*n* = 20; *, *P* < 0.05 vs. WT). (**D**) Representative flow cytometry plots and (**E**) their quantification results showing the reduced uptake of red dye–labeled dead cells in KO macrophages, compared with WT (*n* = 3; *, *P* < 0.05 vs. WT). (**F**) Representative flow cytometry histograms and (**G**) their quantification results showing the reduced surface levels of MERTK in KO macrophages, compared with WT (*n* = 6; *, *P* < 0.05 vs. WT; MFI, mean fluorescence intensity). (**H**) The IL-10 levels in the culture supernatants of KO macrophages were decreased at 12 hours after efferocytosis of dead cells, compared with WT controls (*n* = 12; *, *P* < 0.05 vs. WT).

Considering that SECTM1a is a secreted protein, we herein utilized a global *Sectm1a*-knockout (*Sectm1a*-KO) mouse model to test the above hypothesis. First, we performed in vitro macrophage efferocytosis assays, using BMDMs collected from KO and WT mice. As depicted in Figure 2A, BMDMs were incubated with these dying/dead cells prelabeled with Cell Tracker Deep Red dye for 2 hours, followed by removal of nonengulfed dying/dead cells and staining with F4/80 for immunofluorescence staining (Figure 2, B and C) or BV510-conjugated CD11b (a surface marker of macrophages) for flow cytometry analysis (Figure 2, D and E). Our results showed that KO BMDMs phagocytosed many fewer dying/dead cells than WT macrophages. This suggests that *Sectm1a* deficiency greatly reduces macrophage efferocytosis capacity. In parallel, we measured MERTK levels in these efferocytotic macrophages by flow cytometry and found that cell surface levels of MERTK were significantly decreased in KO BMDMs, compared with WT macrophages (Figure 2, F and G), indicating the impaired efferocytosis. Given that efferocytotic macrophages can release IL-10 to promote injury resolution (13, 14), we then measured IL-10 levels in the supernatants collected from the coculture of BMDMs with dying/dead cells. Our ELISA results showed that KO BMDMs produced less IL-10 than WT macrophages did upon stimulation with dying/dead cells (Figure 2H), further validating the limited efferocytosis in *Sectm1a*-null macrophages. Therefore, the above 3 lines of evidence clearly demonstrate that *Sectm1a* is a critical factor for macrophages to conduct efferocytosis efficiently.

**Figure 3:**
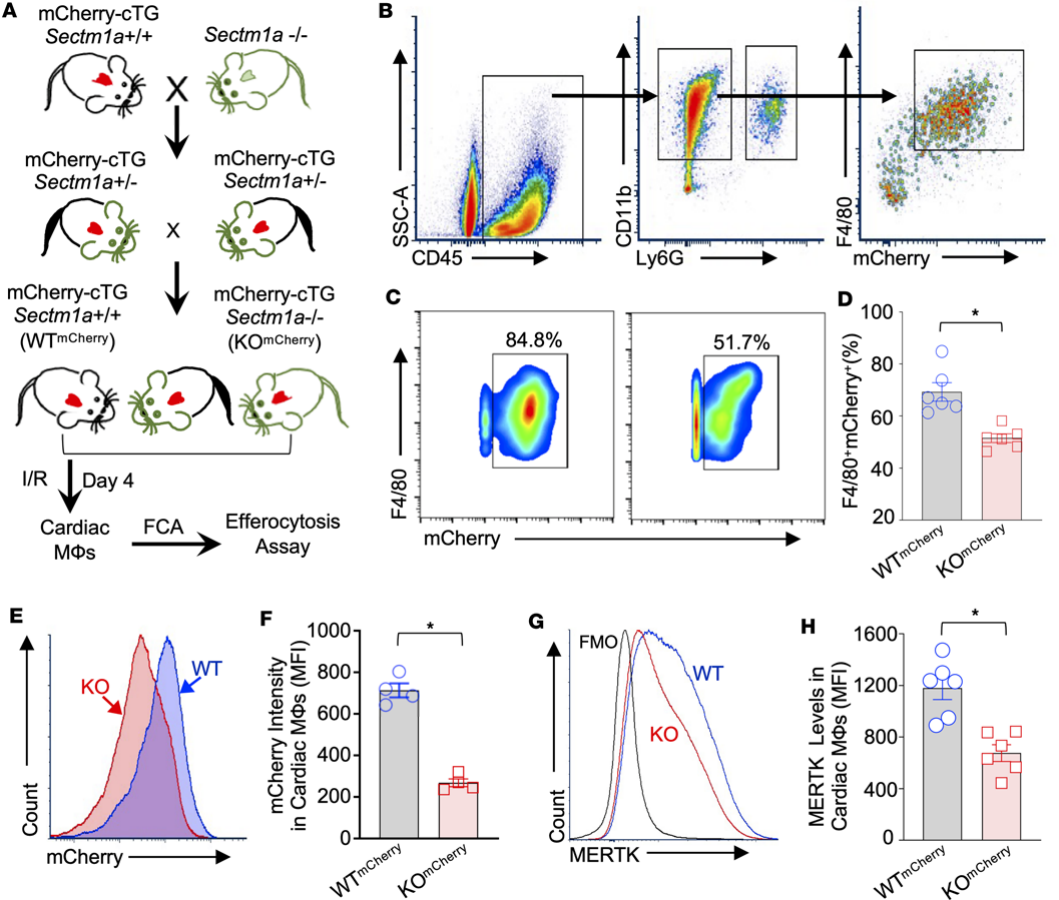
*Sectm1a* deficiency impairs cardiac macrophage efferocytosis in mice after myocardial I/R. (**A**) Schematic illustration for generating heart-specific overexpression of red mCherry in *Sectm1a*-KO mice (KO^mCherry^) and WT mice (WT^mCherry^) and in vivo analysis of cardiac macrophage efferocytosis. (**B**) Gating strategy for flow cytometry analysis of mCherry^+^ in cardiac macrophages. (**C**) Representative flow cytometry plots and (**D**) their quantification of mCherry^+^ macrophages in murine hearts at day 4 after myocardial I/R (*n* = 6; *, *P* < 0.05 vs. WT^mCherry^). (**E**) Representative flow cytometry histograms and (**F**) their quantification of mCherry intensity (MFI) in cardiac macrophages isolated from mice at day 4 post-I/R (*n* = 4; *, *P* < 0.05 vs. WT^mCherry^). (**G**) Representative flow cytometry histograms and (**H**) their quantification of MERTK levels on the surface of cardiac macrophages isolated from mice at day 4 post-I/R (*n* = 6; *, *P* < 0.05 vs. WT^mCherry^). All results are shown as mean ± SEM and analyzed by Student’s *t* test. FMO, fluorescence minus one control.

To determine whether *Sectm1a* deficiency affects macrophage efferocytosis in the I/R hearts, we then generated a mouse model by crossbreeding *α*-*MHC*-mCherry-transgenic (mCherry-cTG *Sectm1a*^+/+^) mice with *Sectm1a*-KO mice, as depicted in Figure 3A. These generated mCherry-cTG *Sectm1a*^+/+^ and mCherry-cTG *Sectm1a*^–/–^ mice are referred to as WT^mCherry^ and KO^mCherry^ mice for short here. First, we isolated peripheral blood mononuclear cells from WT^mCherry^ and KO^mCherry^ mice and analyzed the immune cell populations using flow cytometry analysis according to differentially expressed markers of each immune subset. Our results showed that there was no significant difference in the blood immune cell distributions between WT^mCherry^ and KO^mCherry^ mice (Supplemental Figure 3, A–G). Similarly, immune cell populations in the spleen of WT^mCherry^ and KO^mCherry^ mice also revealed no differences between the 2 groups (Supplemental Figure 4, A–H). Then, we subjected these WT^mCherry^ and KO^mCherry^ mice to in vivo 45-minute myocardial ischemia, via coronary artery occlusion, followed by 4-day reperfusion, when cardiac macrophages were isolated for flow cytometry analysis of efferocytosis, with the gating strategy shown in Figure 3B. We observed that in I/R-injured KO^mCherry^ hearts, macrophages (CD45^+^CD11b^+^F4/80^+^Ly6G^–^) engulfed a lower number of red mCherry-positive CMs than WT^mCherry^ macrophages did (Figure 3, C and D). Consistently, the mCherry intensity histograms also revealed a significant reduction in KO^mCherry^ macrophages, compared with WT control cells (Figure 3, E and F). In addition, we isolated cardiac macrophages from KO^mCherry^ and WT^mCherry^ mice at D4 post-I/R and used confocal microscopy to further validate the decreased mCherry intensity in KO macrophages, compared with WT controls (Supplemental Figure 5, A and B). Concomitantly, lower levels of MERTK were exhibited in the cell surface of these cardiac macrophages collected from KO^mCherry^ mice at D4 post-I/R, compared with control WT^mCherry^ macrophages (Figure 3, G and H). Taken together, these results suggest that macrophage efferocytosis is defective in *Sectm1a*-deficient hearts during I/R.

### Sectm1a deficiency aggravates I/R-induced cardiac apoptosis and injury.

**Figure 4:**
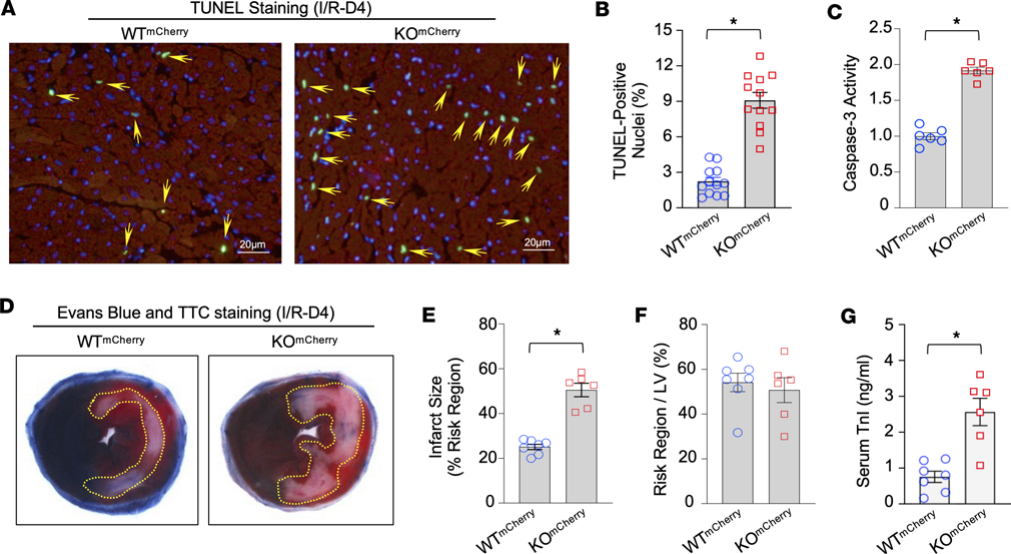
Loss of *Sectm1a* aggravates I/R-induced cardiac cell death. (**A**) Representative images and (**B**) their quantitative results of the TUNEL staining in WT^mCherry^ and KO^mCherry^ hearts after in vivo 45 minutes of left anterior descending artery (LAD) occlusion followed by 4 days of reperfusion (I/R-D4). Green: TUNEL-positive nuclei (pointed by yellow arrows); red: mCherry-expressing cardiomyocytes; blue: DAPI. (*n* = 6 hearts and 2 sections for each heart; *, *P* < 0.05 vs. WT^mCherry^). (**C**) Caspase-3 activity was measured in heart homogenates of WT^mCherry^ and KO^mCherry^ mice subjected to in vivo myocardial I/R (I/R-D4) (*n* = 6; *, *P* < 0.05 vs. WT^mCherry^). (**D**–**F**) Knockout of *Sectm1a* greatly increased myocardial infarct size after in vivo I/R (I/R-D4). The mouse hearts were stained by 5% Evans blue and 1% triphenyl-tetrazolium chloride (TTC) after in vivo I/R. White/gray: infarct size (yellow dot line inside images); red: area at risk; blue, nonischemic region (*n* = 7 for WT^mCherry^ group, *n* = 6 for KO^mCherry^ group; *, *P* < 0.05 vs. WT^mCherry^). (**G**) The circulating Troponin I (TnI) was measured in the sera collected from WT^mCherry^ and KO^mCherry^ mice at day 4 after myocardial I/R (*n* = 7 for WT^mCherry^ group, *n* = 6 for KO^mCherry^ group; *, *P* < 0.05 vs. WT^mCherry^). All results are shown as mean ± SEM and analyzed by Student’s *t* test.

Knowing that *Sectm1a* deficiency jeopardizes efficient efferocytosis in I/R hearts, we then asked whether cardiac apoptosis and injury are more severe in *Sectm1a*-KO hearts than WT controls after I/R. Using in situ TUNEL staining to detect apoptotic DNA fragmentation, we observed that apoptotic CMs were remarkably accumulated in KO^mCherry^ hearts, compared with WT^mCherry^ hearts, at day 4 post-I/R (Figure 4, A and B). Consistently, the activity of Caspase-3, a crucial death protease well known to be a reliable marker for dying/dead cells (39), was significantly increased by 1.92-fold in KO^mCherry^ hearts in comparison with WT^mCherry^ hearts after I/R (Figure 4C). In addition, we measured myocardial infarct size at day 4 post-I/R and showed that the ratio of the infarct-to-risk region was significantly higher in KO^mCherry^ hearts than that in WT^mCherry^ hearts (Figure 4, D and E) whereas the region at risk between groups was similar (Figure 4F). Finally, we assessed the serum levels of Troponin I (TnI), a biomarker of cardiac injury (40), in these mice at day 4 post-I/R, using an ELISA kit. As shown in Figure 4G, KO^mCherry^ mice exhibited 3.37-fold higher levels of serum TnI, compared with control WT^mCherry^ mice, after myocardial I/R. Collectively, these results indicate that *Sectm1a* deficiency exacerbates I/R-caused cardiac cell death, which should be ascribed to the compromised efferocytosis.

### Sectm1a knockout augments I/R-induced cardiac inflammation, fibrosis, and dysfunction.

**Figure 5:**
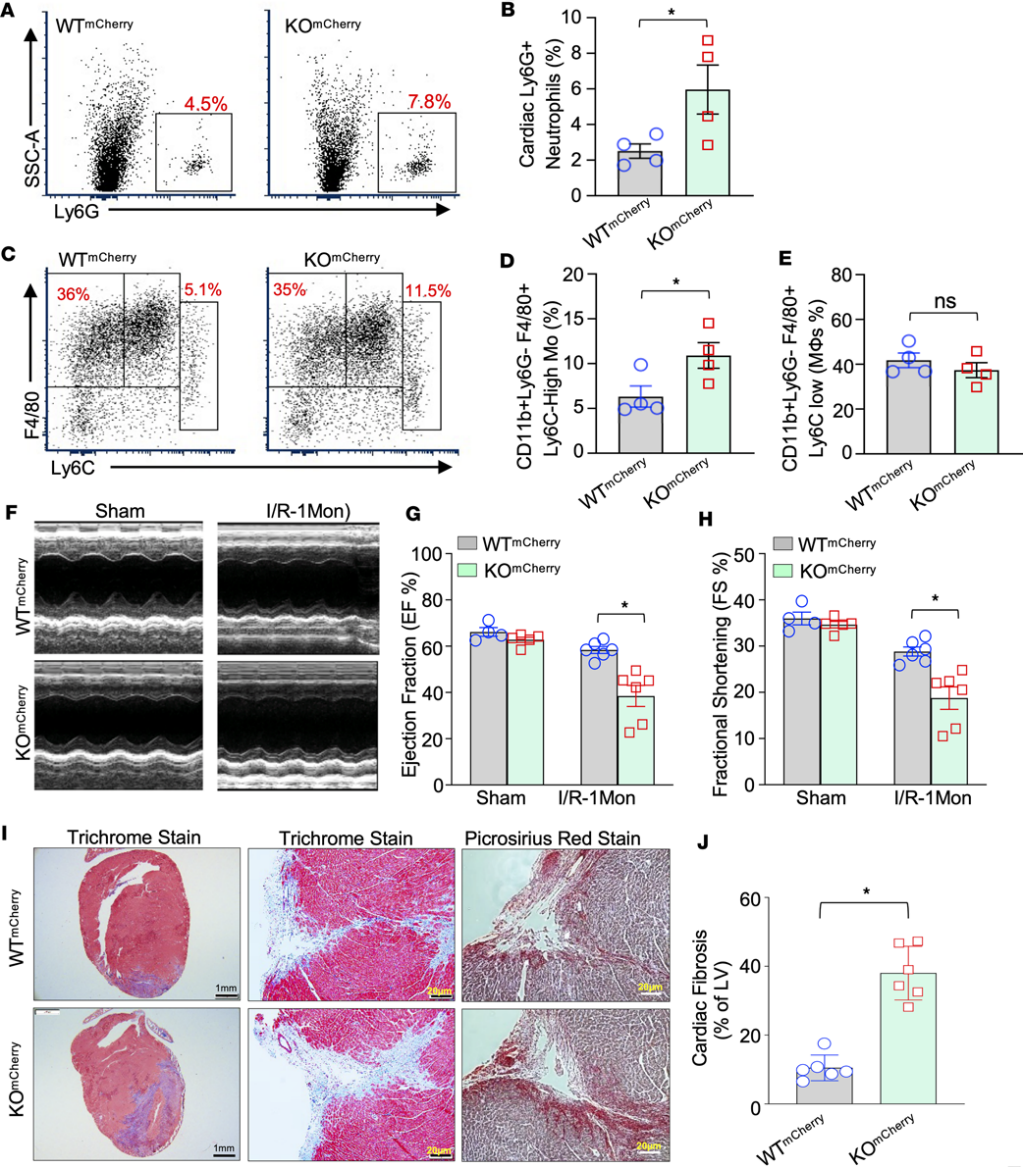
*Sectm1a* deficiency exaggerates I/R-induced cardiac inflammation, fibrosis, and dysfunction. (**A**) Representative flow cytometry plots and (**B**) their quantification results showing the higher number of neutrophils in KO^mCherry^ hearts than WT^mCherry^ hearts at day 7 after myocardial I/R (*n* = 4, *, *P* < 0.05 vs. WT^mCherry^). (**C**) Representative flow cytometry plots and their quantification results of (**D**) total monocytes (CD11b^+^Ly6G^-^F4/80^+^Ly6C^hi^) and (**E**) total macrophages (CD11b^+^Ly6G^–^F4/80^+^ Ly6C^lo^) in murine hearts at day 7 post-I/R (*n* = 4, *, *P* < 0.05 vs. WT^mCherry^). (**F**–**H**) Heart function was analyzed by echocardiography in WT^mCherry^ and KO^mCherry^ mice at 1 month post-I/R (I/R-1Mon). Representative images of M-mode views (**F**), and quantifications of left ventricular ejection fraction (**G**) as well as fraction shortening (**H**) calculated in 4 groups as indicated (*n* = 4–5 for sham groups, *n* = 6 for myocardial I/R group; *, *P* < 0.05 vs. WT^mCherry^). (**I**) Representative images of Masson’s trichrome and Picrosirius red staining and (**J**) their quantification results of cardiac fibrosis in WT^mCherry^ and KO^mCherry^ mice at 1 month after myocardial I/R (*n* = 6; *, *P* < 0.05 vs. WT^mCherry^). Results are presented as mean ± SEM and analyzed by Student’s *t* test (**B**, **D**, **E**, and **J**) or 2-way ANOVA (**G** and **H**).

Considering that increased cell death could stimulate inflammatory response, we next went on to determine the infiltration of inflammatory cells within mouse hearts at day 7 post-I/R, using flow cytometry analysis. Currently, it is well appreciated that neutrophils and monocytes/macrophages with pro-inflammatory phenotypes are typically recruited to I/R hearts (5, 6). Therefore, according to previous reports (28, 29, 41, 42), we characterized cardiac neutrophils as CD45^+^Ly6G^+^ cells, cardiac macrophages as CD45^+^Ly6G^–^CD11b^+^F4/80^+^Ly6C^lo^, and cardiac monocytes as CD45^+^Ly6G^–^CD11b^+^ F4/80^+^Ly6C^hi^, and the gating strategy is shown in Supplemental Figure 6. Please note that total number of CD45^+^ cells was significantly increased by 1.5-fold in KO^mCherry^ hearts at the time point of 7 days post-I/R, compared with control WT^mCherry^ groups (Supplemental Figure 7, A and B). Accordingly, our analysis results showed that the number of neutrophils in KO^mCherry^ hearts was increased by 2.4-fold, compared with WT^mCherry^ groups, after 7-day reperfusion of ischemic hearts (Figure 5, A and B). At the same time point, the total number of monocytes within hearts was significantly elevated by 1.7-fold in KO^mCherry^ mice after cardiac I/R, in comparison with WT^mCherry^ control samples (Figure 5, C and D), while the total numbers of cardiac macrophages revealed similarities between the 2 groups (Figure 5E). Consistently, the expression levels of TNF-α and IL-6 (2 pro-inflammatory cytokines) as well as Cxcl1 (a chemokine for recruiting inflammatory cells) were significantly increased in KO^mCherry^ hearts compared with control WT^mCherry^ hearts after 7-day I/R (Supplemental Figure 7, C–E). Together, these results further corroborate that severe cardiac cell death observed in *Sectm1a* deletion mice after I/R could aggravate the infiltration of inflammatory cells into hearts, leading to the increased cardiac inflammation.

We next measured cardiac function in WT^mCherry^ and KO^mCherry^ mice at 1 month after myocardial I/R, using echocardiography. As shown in Figure 5, F–H, and Supplemental Table 3, I/R-induced cardiac dysfunction was more pronounced in KO^mCherry^ mice, as evidenced by a significant reduction in ejection fraction (EF%) (Figure 5G) and fractional shortening (FS%) (Figure 5H), compared with WT^mCherry^ controls. Given that increased infiltration of immune cells into the myocardium during I/R is a major contributor to cardiac fibrosis (43), we, therefore, performed Masson’s trichrome staining and Picrosirius Red staining in cardiac sections collected from WT^mCherry^ and KO^mCherry^ mice at 1 month after myocardial I/R. As shown in Figure 5, I and J, I/R-treated KO^mCherry^ hearts displayed severe fibrosis to a greater degree than I/R-treated WT^mCherry^ groups. Collectively, these results indicate that depletion of *Sectm1a* in mice deteriorates myocardial I/R-induced adverse cardiac remodeling (e.g., inflammation, dysfunction, and fibrosis).

### RNA-Seq and gene enrichment analysis of Sectm1a-KO macrophages.

**Figure 6:**
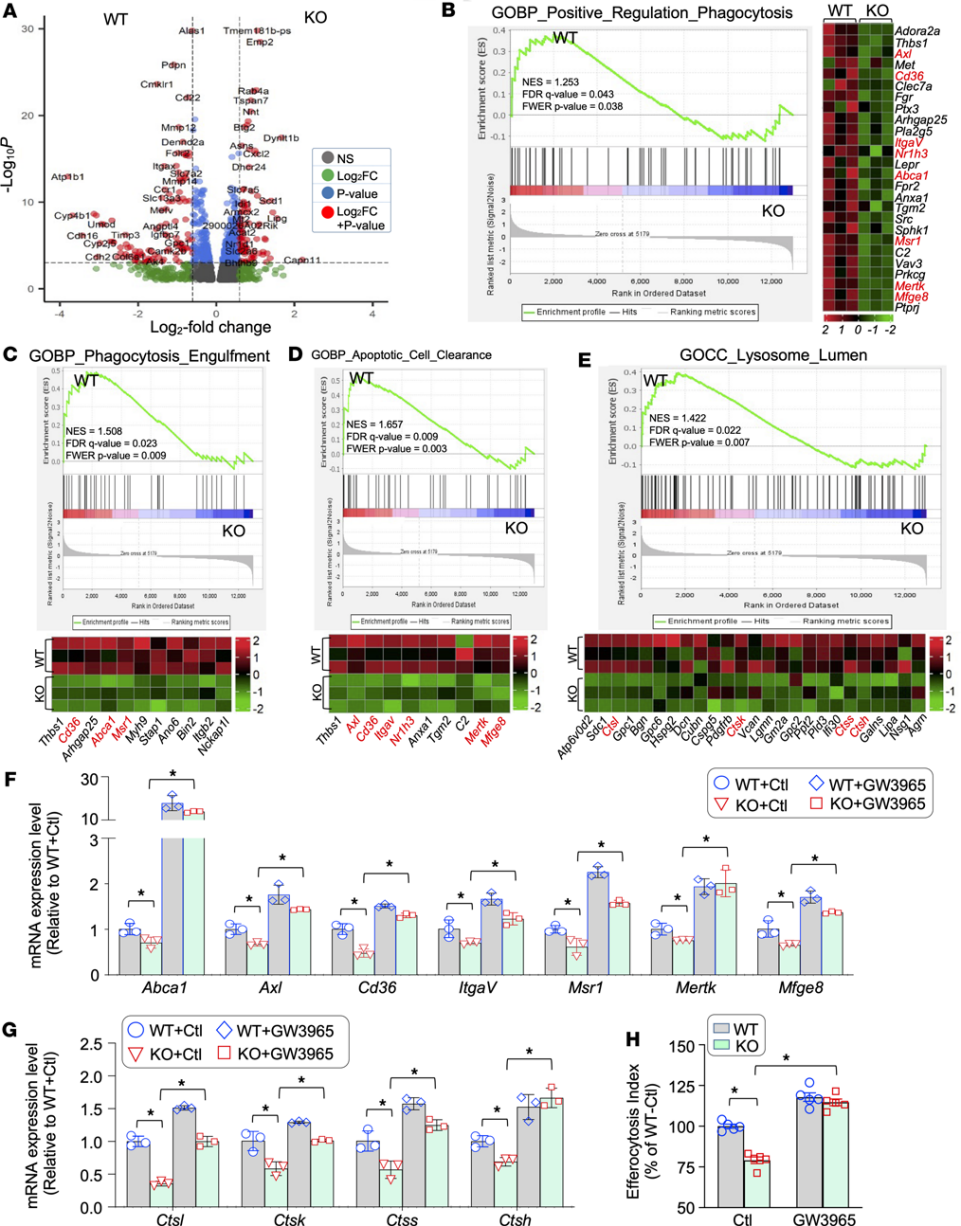
Gene expression profile analysis of KO and WT macrophages. (**A**) Volcano plot of differential gene expression identified by RNA-Seq in KO macrophages relative to WT controls. The *x* axis represents the log_2_ fold-change, and the *y* axis represents the –log_10_ *P* value. The significant genes are depicted in red, indicating that they exhibit both a *P* value (–log10) < 10^–4^ and an absolute log_2_ fold-change > 0.6. (**B**–**E**) Gene set enrichment analysis (GSEA) plots and heatmaps for efferocytosis-related gene sets in WT macrophages compared with KO macrophages. Enriched efferocytosis-related Gene Ontology (GO) terms in genes repressed in KO macrophages, including (**B**) positively regulated phagocytosis, (**C**) phagocytosis engulfment, (**D**) apoptotic cell clearance, and (**E**) lysosome lumen. Green curves indicate enrichment scores. The normalized enrichment score (NES), false discovery rate (FDR) *q* value, and FWER *P* value are indicated within each graph. NES > 1.1 and *P* < 0.05 were considered statistically significant. Representative genes were listed in the heatmap of each enrichment plot. Green and red color indicate down- versus upregulated genes, respectively. The genes written in red beside each heatmap are known to be controlled by *LXRα*. (**F** and **G**) RT-qPCR analysis validates that these efferocytosis-related genes are downregulated in KO macrophages but are upregulated by stimulation with GW3965, an agonist of LXRα (*n* = 3; *, *P* < 0.05). Expression of β-actin was used as the internal control for RT-qPCR. (**H**) Flow cytometry assay showing that the impaired efferocytosis in KO macrophages can be rescued by GW3965 treatment (*n* = 5; *, *P* < 0.05). All data are presented as mean ± SEM and analyzed by 2-way ANOVA (**F** and **H**).

To dissect the potential mechanism underlying the established link between *Sectm1a* and macrophage efferocytosis, we reanalyzed our previous RNA-Seq data generated from *Sectm1a*-KO and WT macrophages (29). To fully capitalize the gene signature of the whole transcriptome, especially to capture lower amplitude signals (e.g., lower fold-changes) from expression variation that would otherwise fall below the signal-to-noise significance cutoff, we first performed unbiased gene set enrichment analysis (GSEA) using whole macrophage transcriptomes. The volcano plot was created using the R package “EnhancedVolcano” (Figure 6A) and showed that there were 2,117 significantly changed genes (FDR < 0.1) out of 12,987 genes used as input. We then performed Gene Ontology (GO) enrichment analysis on differentially expressed genes and focused on GO pathways related to efferocytosis. Our analysis results demonstrated that ablation of *Sectm1a* in macrophages significantly reduced the expression of several sets of genes that are associated with the regulation of endocytosis (Supplemental Figure 8, A and B) and phagocytosis (Figure 6B), phagocytotic engulfment (Figure 6C), apoptotic cell clearance (Figure 6D), and lysosome function (Figure 6E). Notably, those differentially expressed genes include *Abca1*, *Axl*, *Cd36*, *ItgaV*, *Msr1*, *Mertk*, and *Mfge8*, which have been well characterized to play an essential role in macrophage efferocytosis for recognizing, binding, and engulfing apoptotic cells (44–46). Furthermore, transcriptional levels of cathepsin family proteins, including *Ctsl*, *Ctsk*, *Ctss*, and *Ctsh*, were greatly decreased in KO macrophages, compared with WT (Figure 6E). Given that *Ctsl*, *Ctsk*, *Ctss*, and *Ctsh* are lysosomal enzymes known to be responsible for protein degradation in lysosomes (47), it could be inferred that depletion of *Sectm1a* not only suppresses the engulfment of dying/dead cells but also restrains lysosomal degradation of these cell debris/corpses.

In addition, we noticed that *Nr1h3* was significantly downregulated in KO macrophages, compared with in WT macrophages (Figure 6, B and D). *Nr1h3* is usually referred to as *LXRα*, a transcription factor that controls many genes’ expression, including efferocytosis-related genes (i.e., *Abca1*, *Axl*, *Cd36*, *ItgaV*, *Msr1*, *Mertk*, and *Mfge8*) (45). Hence, these data suggest that ablation of *Sectm1a* disrupts the *LXRα*-controlled signaling cascades and, consequently, impairs macrophage efferocytosis capacity. Along this line, we then asked whether activation of *LXRα* could rescue the expression of its controlled genes in KO macrophages and restore efferocytosis capacity. To this end, we treated WT and KO macrophages with a *LXRα* agonist, GW3965, for 12 hours, followed by RT-qPCR analysis and efferocytosis assay. As shown in Figure 6F, consistent with RNA-Seq data above, knockout of *Sectm1a* in macrophages significantly downregulated expression of efferocytotic genes, including *Abca1*, *Axl*, *Cd36*, *ItgaV*, *Msr1*, *Mertk*, and *Mfge8*, compared with those in WT macrophages. Remarkably, expression levels of these efferocytotic genes were significantly elevated in KO macrophages upon treatment with GW3965, compared with those without GW3965 exposure (Figure 6F). Similar findings were also observed for those lysosomal enzyme genes (e.g., *Ctsl*, *Ctsk*, *Ctss*, and *Ctsh*) in KO macrophages without or with GW3965 treatment (Figure 6G). Accordingly, the addition of GW3965 to KO macrophages greatly restored their efferocytosis capacity (Figure 6H). Taken together, these data implicate that defective efferocytosis in *Sectm1a*-KO macrophages is largely ascribed to the disruption of the *LXRα*-controlled pathway.

### SECTM1A regulates LXRα-mediated efferocytosis via GITR.

**Figure 7:**
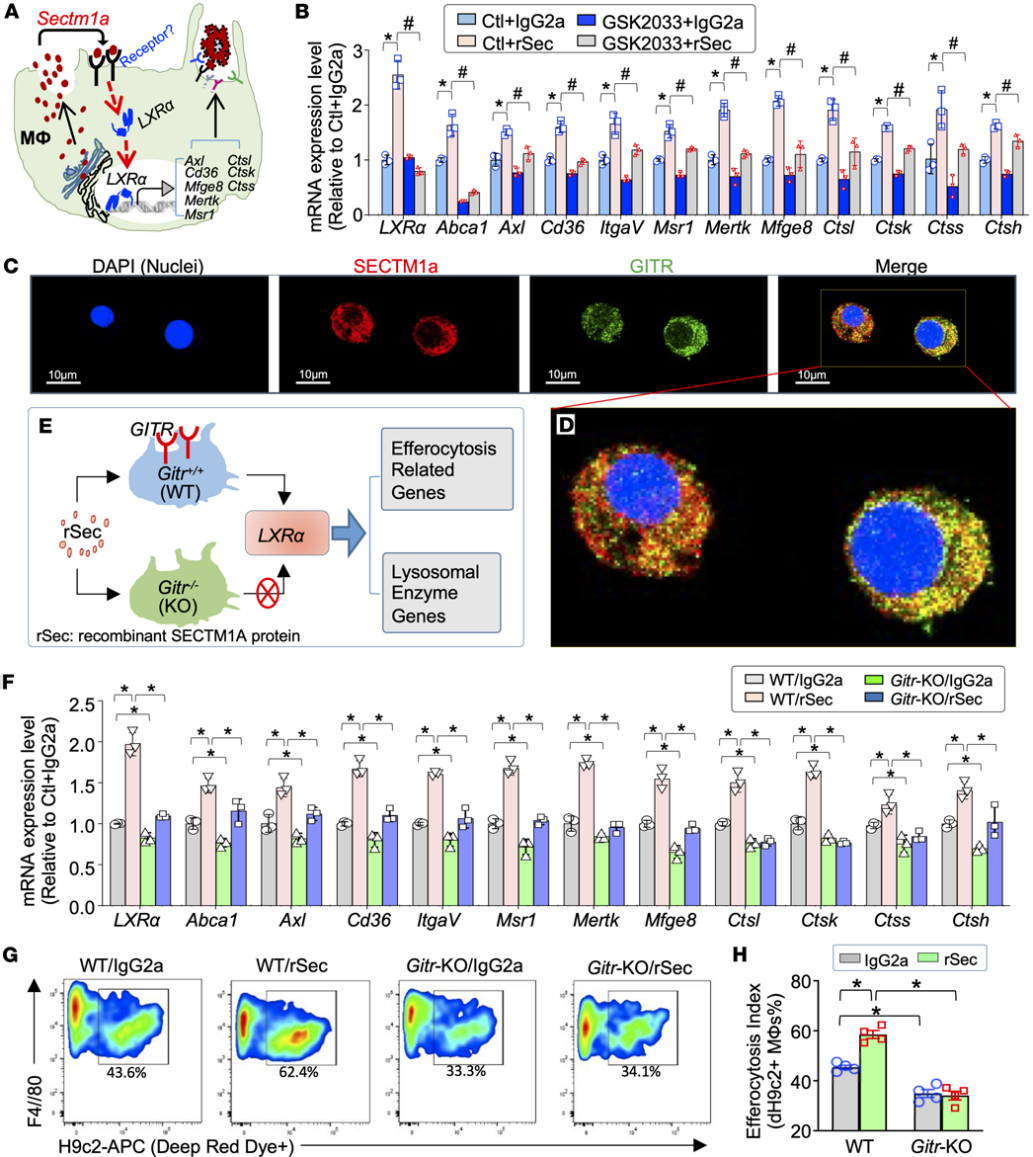
*Sectm1a*-mediated macrophage efferocytosis is largely dependent on the *Gitr*/*LXRα* signaling pathway. (**A**) Cartoon scheme depicting work hypothesis that macrophage-enriched *Sectm1a* has autocrine effects in the activation of *LXRα* signaling cascades including efferocytotic receptor genes and lysosomal enzyme genes. (**B**) RT-qPCR analysis showing that the exogenous addition of recombinant SECTM1A protein (rSec) to macrophages can upregulate the expression of *LXRα* and its downstream genes that are related to efferocytosis, whereas such elevations can be offset by pretreatment with GSK2033, an antagonist of LXRα (*n* = 3; *, *P* < 0.05 vs. the group of Ctl + IgG2a; #, *P* < 0.05 vs. the group of Ctl + rSec). (**C**) Representative image of co-immunofluorescence staining for SECTM1A (red) and GITR (green) in murine macrophages (blue, DAPI for nuclei). Scale bar: 10 μm. (**D**) Magnified image from the yellow frame insert within **C**. (Original magnification: ×400.) (**E**) Schematic illustration of the experiment design and procedure for (**F**) determining the role of *Gitr* in rSec-mediated activation of *LXRα* signaling, measured by RT-qPCR (*n* = 3; *, *P* < 0.05). (**G**) Representative flow cytometry plots and (**H**) their quantification results showing the enhanced efferocytosis in rSec-treated macrophages but being blocked by knockout of *Gitr* (*n* = 4; *, *P* < 0.05). All data are presented as mean ± SEM and analyzed by 2-way ANOVA (**B**, **F**, and **H**). Expression of β-actin was used as the internal control for RT-qPCR.

Given that *Sectm1a* can be secreted into the extracellular environment, we next asked whether exogenous addition of rSec to macrophages could activate LXRα signaling, leading to enhanced macrophage efferocytosis (Figure 7A). To this end, we treated BMDMs with rSec (200 ng/mL) or control IgG2a for 12 hours and then collected cells to isolate total RNAs for RT-qPCR analysis of efferocytosis-related genes and lysosome enzyme genes. In addition, to determine whether rSec-elicited effects on these efferocytosis/lysosomal genes’ expression is dependent on the activation of *LXRα*, we pretreated BMDMs with GSK2033 (2 μM), an antagonist of *LXRα*, for 12 hours, followed by addition of rSec and RT-qPCR assay. As shown in Figure 7B, treatment of BMDMs with rSec did significantly upregulate *LXRα* expression, in comparison with control-treated cells. Consequently, expression levels of *LXRα*-related downstream genes, including *Abca1*, *Axl*, *Cd36*, *ItgaV*, *Msr1*, *Mertk*, *Mfge8*, *Ctsl*, *Ctsk*, *Ctss*, and *Ctsh*, were markedly elevated in rSec-treated macrophages, compared with IgG2a cells (Figure 7B). As expected, blockade of LXRα in BMDMs with its antagonist GSK2033 significantly attenuated such rSec-mediated upregulation of the efferocytotic and lysosomal genes mentioned above (Figure 7B).

We next went on to investigate how *Sectm1a* activates *LXRα* in macrophages. Previously, we showed that SECTM1A interacts with membrane receptor GITR during the macrophage phagocytosis of bacteria (27). However, whether *Sectm1a* activates *LXRα* via *Gitr* in macrophages remains unclear. To add a piece to this puzzle, we first performed co-immunostaining of macrophages with anti-SECTM1A and anti-GITR antibodies. As shown in Figure 7, C and D, SECTM1A was partially colocalized with GITR at the surface of macrophages. We then asked whether *Gitr* was required for *Sectm1a*-mediated activation of the *LXRα* signaling cascades in macrophages. To answer this question, we collected BMDMs from *Gitr*-KO and WT mice, followed by addition of rSec (200 ng/mL) or control IgG2a for 12 hours, followed by RT-qPCR assays (Figure 7E). Our analysis results showed that exogenous addition of rSec did remarkably upregulate expression of *LXRα* in WT macrophages but not in *Gitr*-KO macrophages (Figure 7F). Accordingly, the expression levels of *LXRα*-downstream genes, including efferocytosis-related (e.g., *Abca1*, *Axl*, *Cd36*, *ItgaV*, *Msr1*, *Mertk*, and *Mfge8*) and lysosomal enzyme genes (e.g., *Ctsl*, *Ctsk*, *Ctss*, and *Ctsh*), were significantly elevated in WT macrophages rather than in *Gitr*-KO macrophages (Figure 7F). Please note that without rSec treatment, *LXRα* and its downstream signaling cascades were greatly inactivated in *Gitr*-KO macrophages, compared with WT (Figure 7F). This could be interpreted that naturally released SECTM1A from macrophages may have autocrine effects on the activation of *Gitr*/*LXRα* signaling in WT macrophages, further validating above data that *Sectm1a*-mediated activation of *LXRα* signaling is through its interaction with GITR.

Last, we performed an efferocytosis assay in these rSec-treated *Gitr*-KO and WT macrophages using flow cytometry. Our results demonstrated that rSec was able to promote efferocytosis capacity in WT macrophages but not in *Gitr*-KO cells (Figure 7, G and H, and Supplemental Figure 9, A and B). Indeed, in the absence of exogenous rSec, *Gitr*-KO macrophages exhibited defective efferocytosis capacity, compared with WT macrophages (Figure 7, G and H, and Supplemental Figure 9, A and B). Taken together, these data establish a link of *Sectm1a* to *LXRα* via *Gitr* for macrophage efferocytosis.

### Therapeutic effects of rSec for the repair of I/R hearts through enhanced cardiac macrophage efferocytosis.

**Figure 8:**
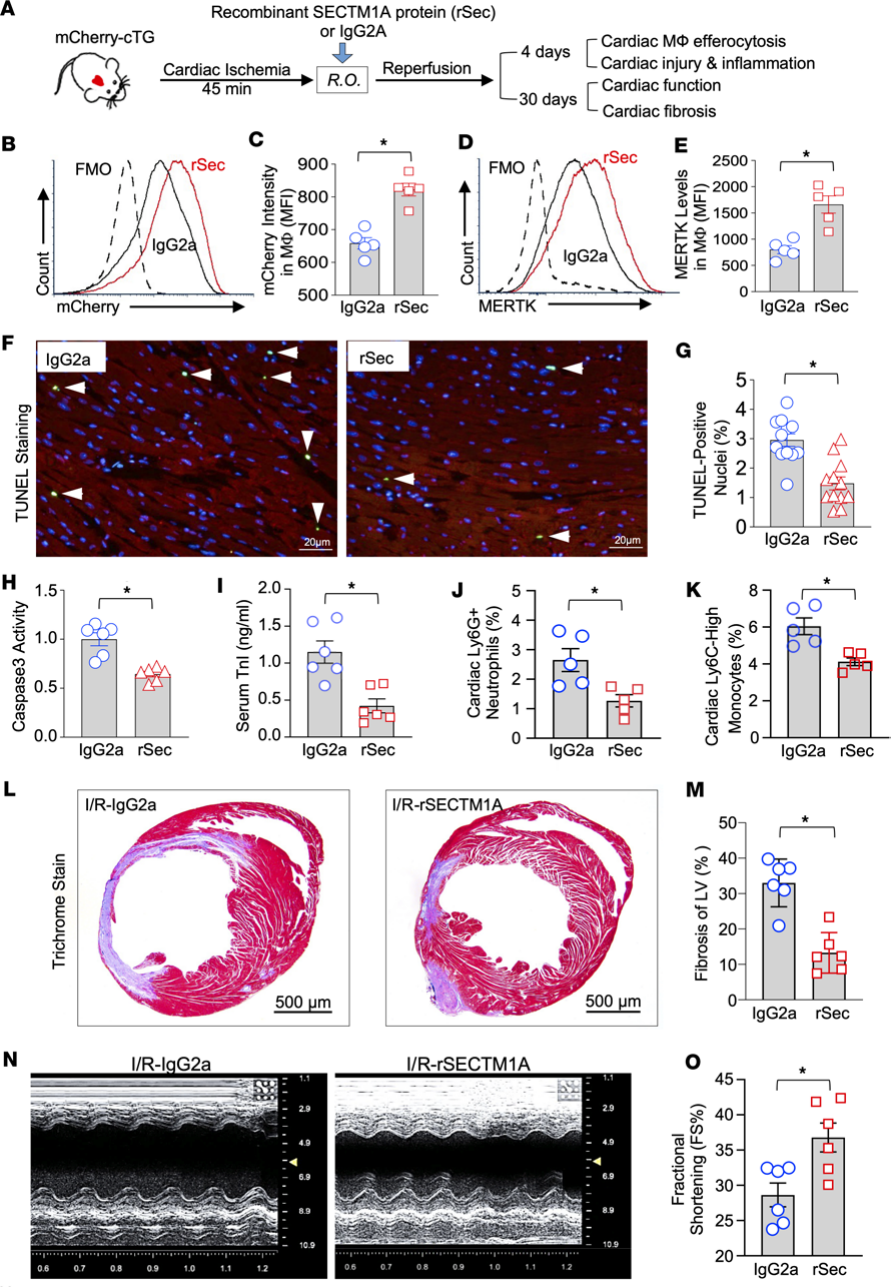
rSec protects against myocardial I/R injury by promoting cardiac macrophage efferocytosis. (**A**) Schematic illustration of the experimental procedure. (**B**) Representative flow cytometry histograms and (**C**) their quantification results showing the increased red mCherry intensity in cardiac macrophages of rSec-treated mice, compared with control mice (*n* = 5; *, *P* < 0.05). (**D**) Representative flow cytometry histograms and (**E**) their quantification results of MERTK levels on the surface of macrophages (*n* = 5; *, *P* < 0.05). (**F**) Representative images and (**G**) quantitative results of the TUNEL staining in rSec-treated and control mouse hearts after in vivo 45 minutes of LAD occlusion followed by 4 days of reperfusion (I/R-D4). Green: TUNEL-positive nuclei (pointed by white arrows); red: mCherry-expressing cardiomyocytes; blue: DAPI. (*n* = 6 hearts and 2 sections for each heart; *, *P* < 0.05.) (**H**) Caspase-3 activity was measured in heart homogenates of rSec-injected and control-treated mice subjected to in vivo myocardial I/R (I/R-D4) (*n* = 6). (**I**) The circulating Troponin I (TnI) was measured in the sera collected from rSec-injected and control-treated mice at day 4 post-I/R (*n* = 6; *, *P* < 0.05). The numbers of cardiac Ly6G^+^ neutrophils (**J**) and cardiac Ly6C-high monocytes (**K**) were measured in rSec-injected and control-treated mice at day 4 post-I/R by flow cytometry assay (*n* = 5; *, *P* < 0.05). (**L**) Representative images of Masson’s trichrome and (**M**) their quantification results of cardiac fibrosis in rSec-injected and control-treated mice at 1 month post-I/R (*n* = 6; *, *P* < 0.05). (**N**) Representative echocardiography images and (**O**) quantification results of myocardial contractile function in rSec-injected and control-treated mice at 1 month post-I/R (*n* = 6; *, *P* < 0.05). All data are presented as mean ± SEM and analyzed by Student’s *t* test. R.O., retro-orbital.

To further test whether rSec has potential therapeutic effects in the repair of I/R-induced myocardial injury, we subjected mCherry-cTG mice to cardiac ischemia for 45 minutes, then injected (retro-orbital) rSec (300 ng/g) into mice prior to reperfusion (Figure 8A). Four days after myocardial I/R, we isolated cardiac macrophages (CD45^+^CD11b^+^F4/80^+^) from rSec-injected and control IgG2a-treated mice, followed by flow cytometry analysis to measure the contents of red mCherry^+^ myocytes that were engulfed by cardiac macrophages. As shown in Figure 8, B and C, administration of cardiac I/R mice with rSec significantly promoted cardiac macrophage efferocytosis, as evidenced by higher amount of mCherry^+^ macrophages in rSec-treated I/R hearts, compared with that in IgG2a-treated I/R hearts. Consistently, the surface levels of MERTK on cardiac macrophages isolated from rSec-injected I/R mice were remarkably increased by 2.1-fold, in comparison with control cardiac macrophages (Figure 8, D and E). Next, we went on to determine I/R-induced cardiac apoptosis by TUNEL staining and caspase-3 activity assays. Our results showed that treatment of cardiac I/R mice with rSec decreased cardiac apoptosis to a greater degree, as evidenced by a significantly lower number of apoptotic CMs (Figure 8, F and G) and 36% reduction of caspase-3 activity (Figure 8H), compared with those in control-treated I/R hearts. In addition, I/R-induced cardiac injury, measured by serum TnI levels, was markedly attenuated by 63% in rSec-treated mice, compared with controls (Figure 8I). Furthermore, we analyzed cardiac inflammation by measuring the total numbers of cardiac neutrophils (CD45^+^Ly6G^+^) and monocytes (CD45^+^Ly6G^–^CD11b^+^Ly6C^hi^). The results of flow cytometry analysis showed that both numbers of neutrophils (Figure 8J) and monocytes (Figure 8K) were significantly decreased in the rSec-treated I/R hearts, compared with IgG2a-treated I/R hearts. Accordingly, pro-inflammatory mediators (TNF-α, IL-6, and Cxcl1) were remarkably downregulated in rSec-treated I/R hearts, compared with controls (Supplemental Figure 10, A–C). Taken together, these data indicate that treatment of cardiac I/R mice with rSec could facilitate macrophage efferocytosis to efficiently clear dying/dead CMs from I/R hearts, leading to reduced cardiac inflammation.

As expected, treatment of myocardial I/R mice with rSec remarkably attenuated cardiac fibrosis, when compared with IgG2a-treated mice (Figure 8, L and M). Last, using echocardiography, we assessed myocardial contractile function and observed that left ventricular FS% and EF% were significantly increased by 1.3-fold and 1.2-fold, respectively, in these rSec-injected mice, compared with control-treated mice at 1 month post-I/R (Figure 8, N and O, and Supplemental Table 4). Together, these translational data suggest that administration of rSec could repair myocardial I/R injury by boosting cardiac macrophage efferocytosis to remove dying/dead CMs and thereby suppressing cardiac inflammatory response.

## Discussion

In the present study, we have identified *Sectm1a* as a previously unrecognized regulator of macrophage efferocytosis in the myocardium to clear excessive dying/dead cells during I/R. Our data presented here demonstrate that human *SECTM1* and its mouse homolog *Sectm1a* both are mostly enriched in cardiac macrophages, compared with other cardiac cell types, which play an indispensable role in macrophage efferocytosis. Accordingly, knockout of *Sectm1a* jeopardizes macrophage efferocytosis, leading to increased apoptotic CMs, augmented cardiac inflammation, fibrosis, and dysfunction in mouse I/R hearts. By contrast, administration of rSec remarkably enhances macrophage efferocytosis, mitigates cardiac adverse remodeling during I/R, and consequently, repairs cardiac I/R injury. The underlying mechanism study reveals that macrophage *Sectm1a* may have autocrine effects and stimulates *Gitr*/*LXRα* signaling cascades to promote the expression of efferocytotic receptor genes for engulfment of apoptotic cells and lysosomal enzyme genes for degradation of engulfed cell debris.

Over the past decades, tremendous effort has been spent on how to regenerate de novo CMs, how to reduce CM apoptosis and fibrosis, as well as how to increase cardiac angiogenesis for the repair of cardiac I/R injury (2–4). However, less attention has been paid to efficient clearance of massive cardiac cell death produced during myocardial I/R. As a matter of fact, recent studies have indicated that macrophage efferocytosis is impaired in the I/R heart, leading to the excessive accumulation of dead cells, thereby causing secondary necrosis to the I/R heart (18–20). In addition, nonengulfed apoptotic CMs may leak their intracellular contents, resulting in danger signaling and inflammation. In contrast, cardiac macrophages during efferocytosis can secrete antiinflammatory cytokines (i.e., TGF-β and IL-10), thereby dampening I/R-triggered cardiac inflammation and preventing/retarding cardiac adverse remodeling (11–13). Hence, enhancing macrophage efferocytosis could rescue such damage and restore cardiac function after I/R. Consistent with this viewpoint, several groups recently did show that increased efferocytosis of apoptotic CMs is able to resolve cardiac inflammation, reduce cardiac fibrosis, increase angiogenesis, and improve cardiac function after myocardial I/R in mice through modification of efferocytotic receptors (i.e., MERTK) (16, 21–23) and other factors (MFEG8, VEGFC, S100A9) (17, 48, 49). Along this line, we elucidate in this study that SECTM1A, a poorly characterized secreted transmembrane protein, is a critical piece for the macrophage efferocytosis puzzle and further bolsters the importance of efficient macrophage efferocytosis in the repair of myocardial I/R injury. Moreover, our data presented in this study show that *Sectm1a*-KO mice exhibit more sensitivity to I/R-induced cardiac damage due to the defective cardiac macrophage efferocytosis, compared with WT. This finding implies that reduction of *SECTM1*, a homolog to murine *Sectm1a*, observed in cardiac macrophages of human patients with DCM, would impair macrophage efferocytosis and contribute to heart failure. In addition, we observed that mouse Sectm1a was downregulated in cardiac macrophages at D1 post-myocardial I/R, but significantly upregulated at D4 and D7 post-I/R (Figure 1F). This could be interpreted by the acute massive inflammation initiated at the early phase of cardiac I/R, as it has been reported that the inflammatory condition can significantly reduce the expression of Sectm1/a in both human monocytes and murine macrophages (29, 30). At the later phase of I/R, cardiac inflammation is mitigated by macrophage efferocytosis, which is consistent with the increased levels of Sectm1a in cardiac macrophages. Indeed, our in vitro data also reveal that expression levels of Sectm1a were significantly increased in macrophages upon exposure to dead/dying cells (Figure 1I). Collectively, our study may provide an important mechanism underlying the defective macrophage efferocytosis that occurs in human failing hearts.

Regarding Sectm1a-mediated macrophage efferocytosis to remove dying/dead CMs from I/R hearts, it would be questioned whether Sectm1a regulates uptake of the infiltrated dying/dead immune cells from I/R hearts. Given that neutrophils are the first to infiltrate into the I/R heart, and they are terminally differentiated and have a short life span due in part to being easily apoptotic (50), we therefore measured total cardiac neutrophils (CD45^+^Ly6G^+^; Supplemental Figure 11, A and B) and engulfed neutrophils by cardiac macrophages (CD45^+^CD11b^+^F4/80^+^Ly6G^+^; Supplemental Figure 11, C and D) in mouse hearts at the time point of D1 post-I/R. Next, we calculated the ratio of engulfed to total neutrophils as the relative efferocytosis of infiltrated neutrophils by cardiac macrophages (Supplemental Figure 11, E and F). In parallel, we also measured total cardiac macrophages using the gating strategy shown in Supplemental Figure 6 and revealed no difference between 2 groups, whereas total number of monocytes was significantly increased in *Sectm1a*-KO hearts, compared with WT hearts at D1 post-I/R (Supplemental Figure 12, A–D). Our results indicate that loss of Sectm1a impairs macrophage efferocytosis of infiltrated neutrophils in I/R hearts (Supplemental Figure 11F), which may partially contribute to the augmented cardiac inflammation in KO I/R mice.

As for the molecular mechanism underlying the enhanced macrophage efferocytosis for the repair of damaged heart, a few studies have been reported and mostly focus on macrophage surface receptors and mitochondrial function (10–13, 51). While significant progress has been made in defining 3 steps involved in macrophage efferocytosis of apoptotic cells including sensing through find-me signals, recognizing/binding through eat-me signals, and internalizing/degrading (10, 12, 13), how to boost cardiac macrophage efferocytosis during myocardial I/R remains obscure. Interestingly, several nuclear receptors, such as peroxisome proliferator–activated receptors, LXRs, retinoic acid receptor, retinoid X receptor, vitamin D receptor, and glucocorticoid receptor, have been reported to orchestrate macrophage efferocytosis through the transcriptional control of key genes of apoptotic cell recognition and internalization, such as *Abca1*, *Cd36*, *Mertk*, *Axl*, *C1qa*, *Mfge8*, *ItgaV*, *Msr1*, and *Tgm2* (45). Nonetheless, how to intrinsically activate these nuclear receptors in cardiac macrophages remains largely unknown. In this study, we believe we provide the first evidence showing that expression levels of *LXRα* and its downstream efferocytosis-related genes (e.g., *Abca1*, *Axl*, *Cd36*, *ItgaV*, *Msr1*, *Mertk*, and *Mfge8*) and lysosomal enzyme genes (e.g., *Ctsl*, *Ctsk*, *Ctss*, and *Ctsh*) are reduced in *Sectm1a*-KO macrophages, whereas they are increased in rSec-treated macrophages, compared with the respective control cells, suggesting the positive correlation of *Sectm1a* with *LXRα*. Furthermore, using LXRα agonist or antagonist to treat *Sectm1a*-null or *Sectm1a*-primed macrophages, we elucidate that cardiac macrophage–enriched *Sectm1a* is an intrinsic factor to regulate *LXRα* activation and, consequently, cardiac macrophage efferocytosis.

Given that SECTM1A is a secreted transmembrane protein (25) and LXRα is a nuclear receptor (45), we then went on to investigate how *Sectm1a* activates *LXRα* in macrophages. Whereas our previous study identified SECTM1A as a GITR ligand that plays a critical role in regulating macrophage phagocytosis to eradicate infected bacteria (27), it remains unclear whether there is a connection between *Sectm1a*/*Gitr* and *LXRα* in macrophages. Our data presented in this study demonstrate that rSec could upregulate the expression of *LXRα* and its downstream genes in WT macrophages but not in *Gitr*-KO macrophages. Functionally, rSec-mediated augmenting of macrophage efferocytosis is offset when GITR is null in macrophages. We realize that our study did not precisely define how *Gitr* affects *LXRα* activation in macrophages, and such analysis falls outside the scope and intent of this report. However, our study presented here fills in a knowledge gap by adding an important piece of *Sectm1a*/*Gitr* to the *LXRα* signaling puzzle.

There are several limitations to this study. First, we utilized a global *Sectm1a*-KO mouse model instead of tissue-specific KO mice. Considering SECTM1A is a secreted protein that may have autocrine effects, the macrophage *Sectm1a*-specific KO mouse model would be unlikely to provide clear and accurate results for discerning the role of *Sectm1a* in macrophage efferocytosis. Nonetheless, while Sectm1a is highly enriched in cardiac macrophages, it is also expressed in CMs, ECs, neutrophils, dendritic cells, and other cell types (30–32). Therefore, we cannot exclude possible contributions of other cell types to the in vivo phenotypic changes displayed in *Sectm1a*-KO mice upon cardiac I/R. For example, loss of Sectm1a in neutrophils and dendritic cells may also impair their efferocytosis capacity, which could contribute partially to cardiac injury exhibited in *Sectm1a*-KO mice upon myocardial I/R. In addition, it remains unclear whether loss of Sectm1a could directly affect I/R-triggered CM apoptosis/necrosis. Second, one would argue that expression levels of *Sectm1a* are significantly increased in cardiac macrophages during myocardial I/R, why does rSec have therapeutic effects for the repair of cardiac I/R injury? Indeed, at early phase (D1) of cardiac I/R, the circulating level of *Sectm1a* is reduced whereas it is increased at a later phase (D7) of cardiac I/R (Figure 1G). Such an elevation of *Sectm1a* in mice at the later phase of myocardial I/R should be a compensatory protective mechanism to intrinsically enhance cardiac macrophage efferocytosis.

In conclusion, the present study provides the evidence showing that *Sectm1a* is essential for cardiac macrophages to carry out efficient efferocytosis in the I/R heart. Mechanistically, we identify the *Sectm1a*/*Gitr*/*LXRα* axis as a link to the regulation of cardiac macrophage efferocytosis. These findings presented in this work greatly advance our knowledge on the mechanism underlying the defective macrophage efferocytosis that occurs in the I/R heart. Most importantly, our data clearly define *Sectm1a* as a potential therapeutic agent for the treatment of cardiac I/R injury through enhancing cardiac macrophage efferocytosis.

## Methods

### Sex as a biological variable.

Our study examined male and female animals, and similar findings were reported for both sexes.

### Mice.

WT, *α-MHC*-mCherry transgenic [Tg (Myh6*-mCherry) 2Mik/J, Strain 021577], and global *Gitr*-KO (*Tnfrsf18^tm1Ppp^*/CptJ, Strain 030648) mice were purchased from The Jackson Laboratory. The global *Sectm1a*-KO mouse model was generated using a CRISPR/Cas9 system and detailed in our previous publication (29). The *α-MHC*-mCherry transgene contains a cardiac specific alpha myosin heavy-chain (*Myh6*) promoter that drives mCherry expression specifically in CMs. All these mice were on the C57BL/6 background and bred in the Division of Laboratory Animal Resources at the University of Cincinnati Medical Center. Male and female mice between 6 and 10 weeks of age were used for experiments in a sex-matched manner.

### Myocardial I/R in vivo and infarct size analysis.

In vivo myocardial I/R surgery was performed as described in our previous publication (9). Briefly, adult (10- to 12-week-old) mice were anesthetized by intraperitoneal (i.p.) injection with a mixture of ketamine (90 mg/kg body weight) and xylazine (20 mg/kg of body weight), then orally intubated and connected to a mouse ventilator (Model 845, Harvard Apparatus). The depth of anesthesia was monitored by toe pinch. Subsequently, a lateral thoracotomy (1.5 cm incision between the second and third ribs) was performed to provide exposure of the left anterior descending coronary artery (LAD), while avoiding rib and sternal resection, retraction, and rotation of the heart. A 6-0 silk suture was placed around the LAD, and a piece of soft silicone tubing (0.64 mm ID, 1.19 mm OD) was placed over the artery. The suture was then tied and tightened for 45 minutes, followed by varying periods of reperfusion. Ischemia was confirmed by visual observation (cyanosis) and continuous ECG monitoring. Sham-operated mice were subjected to the same surgical procedures, except that the suture was passed under the LAD but not tied. After reperfusion, the aorta was cannulated and perfused with 5% Evans blue, and the hearts were rapidly excised and frozen in a –80°C freezer for 4 hours and sliced into 5 slices, which were subsequently incubated in 1% TTC (MilliporeSigma, catalog T8877)] for 30 minutes at 37°C to distinguish the ischemia and infarct myocardium within the area at risk (AAR). The infarct size (IS), AAR, and nonischemic left ventricle (LV) were assessed with image analysis software ImageJ (NIH). The ratio of AAR/LV and IS/AAR was calculated.

### Isolation, culture, and treatment of BMDMs.

BMDMs were isolated and cultured as we described previously (41). Briefly, WT, *Sectm1a*-KO, or *Gitr*-KO mice were euthanized as above, followed by the removal of both hind legs. The skin was removed and then immersed in 75% ethanol for 3 minutes at room temperature, followed by immersing them in PBS for 2 minutes, and then the muscle was removed. Subsequently, bone marrows from the femur and tibia bones were flushed out by using 25G needles filled with a cold, sterile DMEM containing 2% FBS and filtered through a 70 μm and 40 μm cell strainer (SPL Life Sciences), followed by the removal of red blood cells (RBCs) in ammonium-chloride-potassium lysis buffer (BioLegend, catalog 420302) for 5 minutes at room temperature. Next, these bone marrow cells were cultured at 37^o^C in a 5% CO_2_ incubator with a complete culture medium (DMEM supplemented with 20% L929 cell culture supernatant, 10% FBS, and 1% penicillin/streptomycin solution, 10 mM HEPES buffer) and allowed to grow and differentiate into macrophages for 7–10 days.

For the efferocytosis assay, Ad.GFP-transfected or Cell Tracker Deep Red dye-labeled H9c2 cardiac cells were treated with H_2_O_2_ (1 mM) for 2 hours to induce apoptosis. Subsequently, these labeled dying/dead cells were added to BMDMs that were preseeded in 6-well plates at a ratio of 5:1, and incubated for 2 hours, followed by flow cytometry analysis. For the *Sectm1a*/*LXRα* signaling pathway analysis, WT and KO BMDMs were plated in 6-well plates (1 10^6^ cells/well) and treated with GW3965 (2 μM, LXRα agonist, TOCRIS Bioscience, catalog 2474) for 12 hours, followed by efferocytosis assay as described above, or followed by isolation of total RNA for RT-qPCR assay. In parallel, WT BMDMs were pretreated with LXRα antagonist (GSK2033, 2 μM, TOCRIS Bioscience, catalog 5694) or control DMSO (0.005%) for 12 hours. Then we added rSec (200 ng/mL, R&D Systems, catalog 7837-ST) or control IgG2a (R&D Systems, catalog MAB003) for 12 hours, followed by efferocytosis and RT-qPCR assays. For the *Sectm1a*/*Gitr* signaling cascade assay, WT and *Gitr*-KO BMDMs were treated with rSec (200 ng/mL) or control IgG2a for 12 hours, followed by efferocytosis and RT-qPCR measurements, as described above.

### Isolation of mouse cardiac macrophages, ECs, myocytes, and fibroblasts.

Cardiac macrophages were isolated using the MagniSort Mouse F4/80 Positive Selection Kit (Invitrogen, catalog 8802-6863) according to the manufacturer’s instructions. Briefly, mouse hearts were perfused with 15 mL of cold PBS via the LV, and then the heart was thoroughly minced and digested in HBSS with 1 mg/mL Collagenase I (Worthington, catalog LS004196), 1 mg/mL Collagenase II (Worthington, catalog LS004177), and 1 mg/mL Dispase II (MilliporeSigma, catalog D4693). After 45-minute incubation at 37°C with gentle agitation, the digested heart tissue pieces were passed through a 40 μm cell filter (SPL Life Sciences) to obtain a single-cell suspension, followed by centrifugation at 500*g* for 5 minutes at 4°C. The resulting cell pellets were resuspended in 1 mL RBC lysis buffer (BioLegend, catalog 420302), incubated for 3 minutes, and washed with PBS. Subsequently, the cells were incubated with biotinylated F4/80 selection antibodies for 10 minutes, followed by centrifugation at 300*g* for 5 minutes at 4°C. The cell pellets were resuspended in cell separation buffer and incubated with magnetic beads for 10 minutes. The tube containing samples was placed in the magnet for 5 minutes, and the supernatant was discarded. After 3 times of positive selections in the magnet and washing with cell separation buffer, cardiac macrophages were collected and ready for RNA isolation and RT-qPCR assay. Primary cardiac ECs, CMs, and FBs were isolated from mice following the protocol described previously (52).

### Measurement of Sectm1a in the blood/culture supernatants and immunostaining.

Mouse sera were collected at the time points of D1 and D7 after myocardial I/R as well as from sham-operated mice. Culture supernatants were collected from cultured BMDMs in the absence and presence of dead/dying cells for 6 hours. The Sectm1a levels were measured in these mouse sera and culture supernatants using an ELISA kit (Biorbyt, catalog orb566236).

For the immunostaining of Sectm1a, BMDMs were plated in the ibidi μ-Dish 35 mm confocal dishes (Thermo Fisher Scientific, 50-305-807) and fixed with 4% paraformaldehyde for 10 minutes, followed by 3-time washing and blocking with 1% goat serum ((Thermo Fisher Scientific, catalog 50-062Z) for 30 minutes. Subsequently, these BMDMs were incubated with primary antibodies diluted in 1% BSA overnight at 4°C in the dark. The following antibodies and dilutions were used: sheep anti-mouse Sectm1a antibody (NOVUS, catalog AF7837, 1:200) and rabbit monoclonal [CAL61] antibody to mouse GITR (Abcam, catalog ab237725, 1:250). After 5-time washing with PBS-Tween (PBST), these confocal dishes were incubated with secondary antibodies: Alexa Fluor 594–conjugated donkey anti-Sheep IgG (Invitrogen, catalog A-11016, 1:500) and Alexa Fluor 488–conjugated goat anti-Rabbit IgG (Invitrogen, catalog A-11008, 1:500) for 60 minutes at room temperature. After washing with PBST, confocal dishes were mounted with ProLong Diamond Antifade Mountant with DAPI (Invitrogen, catalog P36961). Images were captured with a Leica confocal microscope (Live Microscopy Core; University of Cincinnati, Cincinnati, Ohio).

### RT-qPCR, bioinformatic assay, and GSEA.

Total RNAs were extracted from macrophages and cardiac cells using the RNeasy Kit (QIAGEN, catalog 217004) in accordance with the manufacturer’s instructions. cDNA was synthesized using Superscript II Reverse Transcriptase (Invitrogen, catalog 18080044). Then qPCR was performed in triplicate with the ABI PRISM 7900HT sequence detection system using SYBR green (GeneCopoeia, catalog QP005). The mRNA expression levels were normalized to the housekeeping gene as indicated in the figure legends (*GAPDH* or β*-actin*), and relative fold-change was calculated by the 2^−ΔΔCt^ method. Sequences of all primers used for RT-qPCR are listed in Supplemental Table 1.

RNA-sequencing analysis was performed by the Genomics, Epigenomics, and Sequencing Core at the University of Cincinnati. Total RNA was converted into libraries of double-strand cDNA as a template and sequenced on an Illumina HiSeq 2500, yielding at least 25 million unpaired 51 base reads per sample of high overall quality. The volcano plot was generated using the R package EnhancedVolcano (1.16). The heatmap was created by Complexheatmap (2.16.0), following the normalization and scaling of data within each specified gene set. The normalized counts generated in DESeq2 were used for analysis in GSEA. The following GO gene set databases were utilized: GOBP_POSITIVE_REGULATION_OF_PHAGOCYTOSIS.v2023.1.Hs.gmt, GOBP_POSITIVE_REGULATION_OF_ENDOCYTOSIS.v2023.1.Hs.gmt, GOBP_PHAGOCYTOSIS_ENGULFMENT.v2023.1.Hs.gmt, GOBP_POSITIVE_REGULATION_OF_PHAGOCYTOSIS.v2023.1.Hs.gmt, GOCC_LYSOSOMAL_LUMEN.v2023.1.Hs.gmt, and GOBP_APOPTOTIC_CELL_CLEARANCE.v2023.1.Hs.gmt.

### Flow cytometry analysis of cardiac inflammatory cells and efferocytosis.

Cardiac inflammatory cell phenotype was assessed using flow cytometry as we described previously (29). In brief, cardiac single-cell suspension was prepared as described above and resuspended in flow cytometry sorting buffer (HBSS with 1 mM EDTA, 25 mM HEPES, and 1% FBS) and incubated on ice with Fc-blocking solution (anti-CD16/32, eBioscience, catalog 14-0161-81, 1:100 dilution) for 10 minutes. After washing, cells were stained with primary antibodies (listed in Supplemental Table 2) at 4°C for 30 minutes in the dark. Then cells were washed twice, then fixed in 2% paraformaldehyde for 15 minutes; flow cytometry assay was performed using LSRII Analyzer at the Flow Cytometry Core (Cincinnati’s Children’s Hospital Medical Center), then analyzed with FCS Express software (Dotmatics).

For the in vitro flow cytometry assay of macrophage efferocytosis, BMDMs were incubated with Deep Red dye–labeled dying/dead H9c2 cells for 2 hours (described above), followed by washing away the nonengulfed cells. Subsequently, these BMDMs were collected and blocked with CD16/32 Ab (clone 93) (1:100 dilution; eBioscience, catalog 101302) to prevent the nonspecific binding to Fc receptors. Following blocking, BMDMs were stained with BV510-conjugated anti-mouse CD11b (1:50 dilution; BioLegend, catalog 101245) and underwent flow cytometry assay to measure the engulfed dead cells by macrophages as efferocytotic macrophages.

For the in vivo flow cytometry assay of macrophage efferocytosis in I/R hearts, cardiac single-cell suspension was collected from mCherry-cTG (WT^mCherry^) and mCherry-cTG *Sectm1a*-KO (KO^mCherry^) mice, respectively, and incubated on ice with Fc blocking for 10 minutes as described above. Subsequently, these cells were labeled with Alexa Fluor 488 anti-mouse CD45.2 antibody (BioLegend, catalog 109816, 1:50), PE anti-mouse/human CD11b antibody (BioLegend, catalog 101208, 1:50), APC anti-mouse F4/80 antibody (BioLegend, catalog 123116, 1:50), BV421 anti-mouse Ly6G antibody (BioLegend, catalog 127628, 1:50), and PE/Cyanine7 anti-mouse MERTK (Mer) antibody (BioLegend, catalog 151522, 1:50) for 25 minutes, then fixed with Fixation Buffer (BioLegend, catalog 420801) in the dark for 20 minutes at room temperature, followed by washing 4 times with Dulbecco’s PBS. Finally, the F4/80^+^mCherry^+^ macrophage population was measured by flow cytometry assay as in in vivo efferocytotic macrophages.

### I/R-induced cardiac injury, apoptosis, and fibrosis assays.

For the cardiac injury assay, serum TnI levels were measured in mice at 4 days after myocardial I/R, using a Mouse Troponin I ELISA Kit (Abcam, catalog ab285235). Cardiac apoptosis was assessed by in situ DNA fragmentation and Caspase-3 activity, using a DeadEnd Fluorometric TUNEL kit (Promega, catalog G3250) and a Caspase-3 Colorimetric Assay kit (Abcam, catalog ab39401), respectively, according to their manuals. TUNEL-positive (green) apoptotic nuclei located in cardiomyocytes (red, mCherry) were counted from 5 randomly chosen microscope fields (original magnification, 400×) of the ventricular section and were expressed as a percentage of total nuclei (blue, stained with DAPI) from the same field. Two sections from each heart and 6 hearts for each group were used. Caspase-3 activity was determined in cardiac lysates (200 μg). Fold-change in Caspase-3 activity can be determined by comparing *Sectm1a*-KO group or protein-treated group with the levels of control group. For the measurement of cardiac fibrosis, mouse hearts were collected at 1 month after myocardial I/R and fixed with 4% paraformaldehyde, embedded in paraffin, and cut into 5 μm–thickness sections, followed by the Masson’s trichrome staining or the Picrosirius Red staining. Images of each slide were taken by an Olympus BX41 microscope equipped with charge-coupled device (MagnaFire, Olympus) camera. Fibrosis area and total left ventricular area of each image were measured using Image-Pro Plus (Media Cybernetics Inc.), and the percentage of cardiac fibrosis was calculated as (fibrosis area/total left ventricular area) × 100.

### Myocardial function measurement.

Cardiac function was assessed in 1.5% isoflurane-anesthetized mice by transthoracic echocardiography, using Vevo 2100 ultrasound imaging system with a 40 MHz transducer as we described previously (29). LV end-diastolic (LVIDd) and end-systolic diameters (LVIDs) were measured from M-mode recordings. The percentage of LV FS was calculated as: FS (%) = [(LVIDd − LVIDs)/LVIDd] × 100. LV EF was calculated as: [(LVIDd^3^ – LVIDs^3^)/LVIDd^3^] × 100. All measurements were performed according to the American Society for Echocardiography leading-edge technique standards and averaged over at least 3 consecutive cardiac cycles.

### Statistics.

GraphPad Prism 8 was utilized for statistical analysis. Data were presented as means ± SEM. Comparison between 2 groups was determined by Student’s 2-tailed *t* test. Differences among multiple groups were determined by 1- or 2-way ANOVA where appropriate. *P* < 0.05 was considered statistically significant.

### Study approval.

All animal experiments conformed to the *Guide for the Care and Use of Laboratory Animals* prepared by the National Academy of Sciences, 2011, published by the NIH, and were approved by the University of Cincinnati Animal Care and Use Committee (21-08-06-01).

### Data availability.

The raw data supporting the conclusions of this article will be made available from the corresponding author upon request. Values for all data points in graphs are reported in the Supporting Data Values file. The RNA-Seq data have been deposited in the NCBI Gene Expression Omnibus (GEO) and are accessible through GEO Series accession number GSE253579.

## Author contributions

XW, YL, and HHY designed and performed experiments and analyzed data. WD and JC helped with the bioinformatic analysis of RNA-Seq data. YZ and RA helped with flow cytometry analysis. HM helped with echocardiography analysis. YCH helped with generation of the *Sectm1a*-KO mouse model. TP, SS, YF, and WH helped with experimental design and data analysis and critically reviewed the manuscript. GCF analyzed the results, wrote the manuscript, provided financial and administrative support, and gave final approval of the manuscript.

## Supplementary Material

Supplemental data

Supporting data values

## Version 1 03/08/2024

Electronic publication
