# Supplementary material for: Macrophage-enriched Sectm1a promotes efficient efferocytosis to attenuate ischemia/reperfusion-induced cardiac injury
Source: JCI Insight. 2024 Mar 8;9(5):e173832. doi: 10.1172/jci.insight.173832 (PMC10972593; doi:10.1172/jci.insight.173832)
Supplement: Supplemental data [file jciinsight-9-173832-s079.pdf]

## Supplementary Tables

### Supplemental Table S1: Primer sequences for RT-qPCR

| Primer name | Forward primer (5′-3′) | Reverse primer (5′-3′) | Product length(bp) |
|---|---|---|---|
| *IL-6* | CTGCAAGAGACTTCCATCCAG | AGTGGTATAGACAGGTCTGTTGG | 131 |
| *TNF-a* | CAGGCGGTGCCTATGTCTC | CGATCACCCCGAAGTTCAGTAG | 89 |
| *Cxcl1* | CTGGGATTCACCTCAAGAACATC | CAGGGTCAAGGCAAGCCTC | 117 |
| *Abca1* | AGTGATAATCAAAGTCAAAGGCACAC | AGCAACTTGGCACTAGTAACTCTG | 136 |
| *β-Actin* | TTCCAGCCTTCCTTCTTG | GGAGCCAGAGCA GTAATC | 182 |
| *Axl* | CCAGGACACCCCAGAGGTGCTAAT | TGGTGGACTGGCTGTGCTTGC | 189 |
| *Cd36* | GATGACGTGGCAAAGAACAG | TCCTCGGGGTCCTGAGTTAT | 107 |
| *Ctsh* | TACAACAAGGGCATCATGGA | TTCTTGACGAATGCAACAGC | 98 |
| *Ctsk* | CAGCAGAGGTGTGTACTATG | GCGTTGTTCTTATTCCGAGC | 174 |
| *Ctsl* | TCTCACGCTCAAGGCAATCA | AAGCAAAATCCATCAGGCCTC | 52 |
| *Ctss* | ATAAGATGGCTGTTTTGGATG | TTCTTTTCCCAGATGAGACGC | 164 |
| *ItgaV* | CTCATCGTTTCTATCCCACC | TTGAGTTCCAGCCTTCATCG | 150 |
| *Lxrα* | CCTGATGTTTCTCCTGACTC | TGACTCCAACCCTATCCTTA | 147 |
| *Mertk* | GTGGCAGTGAAGACCATGAAGTTG | GAACTCCGGGATAGGGAGTCAT | 568 |
| *Mfge8* | ATCTACTGCCTCTGCCCTGA | CCAGACATTTGGCATCATTG | 103 |
| *Msr1* | TGGAGGAGAGAATCGAAAGCA | CTGGACTGACGAAATCAAGGAA | 140 |
| *Sectm1a* | CAGTGATGACCTGTAACATCTC | CAAGTATATCCCTGTGTGGTCG | 197 |
| *Sectm1b* | GAGAAGCAGGTAAGAAGCTGGAG | CAGTTCACACCGAAGAACCC | 95 |
| *Sectm1a (Genotyping)* | CATTCTCTCCATACAGGCTGG | CTTGAACTTGGAGCTCCCAC | KO: 127; WT: 211 |

### Supplemental Table S2: Antibodies and Reagents used for flow cytometry analysis

| Reagent name | Manufacturer | Clone | Catalog# | Dilution |
|---|---|---|---|---|
| CD11b (BV510) | Biolegend | M1/70 | 101245 | 1:50 |
| CD11b (BV650) | Biolegend | M1/70 | 101259 | 1: 50 |
| CD11b (Alexa Fluor-594) | Biolegend | M1/70 | 101254 | 1: 50 |
| CD11b-PE | Biolegend | M1/70 | 101208 | 1:50 |
| CD16/32 | eBioscience | 93 | 101302 | 1: 100 |
| CD45 (Alexa Fluor-488) | Biolegend | S18009D | 160306 | 1: 50 |
| CD45.2 (Alexa Fluor-488) | Biolegend | 104 | 109816 | 1: 50 |
| F4/80 (APC) | Biolegend | BM8 | 123116 | 1: 50 |
| F4/80 (BV421) | Biolegend | BM8 | 123132 | 1: 50 |
| F4/80 (PE) | Biolegend | BM8 | 123110 | 1: 50 |
| Ly6C (PE/Dazzle 594) | Biolegend | HK1.4 | 128044 | 1: 50 |
| Ly6G (BV421) | Biolegend | 1A8 | 127628 | 1: 50 |
| Ly6G (APC) | Biolegend | 1A8 | 127614 | 1: 50 |
| MerTK (PE/Cyanine7) | Biolegend | 2B10C42 | 151522 | 1: 50 |
| Fixation Buffer | Biolegend | / | 420801 | / |
| Live/Dead Fixable Blue Dead Cell Stain Kit | Thermo Fisher | / | L34962 | 1: 100 |

## Supplemental Table S3: Echocardiographic measurements of cardiac function in WT and Sectm1a-KO mice at 1-month after cardiac I/R

| Parameter | Units | WT-Sham (n = 4) | KO-Sham (n = 5) | WT-I/R (n = 6) | KO-I/R (n = 6) |
|---|---|---|---|---|---|
| Heart Rate (HR) | bpm | 567 ± 28 | 572 ± 33 | 558 ± 34 | 549 ± 37 |
| Diameter;s | mm | 2.41 ± 0.22 | 2.57 ± 0.17 | 2.86 ± 0.17* | 3.83 ± 0.52 # |
| Diameter;d | mm | 3.75 ± 0.23 | 3.92 ± 0.14 | 4.03 ± 0.29 | 4.65 ± 0.33 # |
| Volume;s | µL | 21.04 ± 4.31 | 26.51 ± 3.54 | 31.41 ± 4.60* | 54.84 ± 4.73 # |
| Volume;d | µL | 60.88 ± 8.65 | 66.10 ± 9.33 | 75.41 ± 3.58* | 93.68 ± 10.86 # |
| Ejection Fraction (EF) | % | 66.16 ± 3.19 | 62.78 ± 2.26 | 58.36 ± 3.33* | 38.49 ± 10.21 # |
| Fractional Shortening (FS) | % | 36.10 ± 2.39 | 34.64 ± 2.43 | 28.82 ± 2.18 * | 17.62 ± 5.20 # |

*, $p < 0.05$, when comparing WT-I/R to WT-Sham; #, $p < 0.05$, when comparing KO-I/R to WT-I/R. Data are presented as Mean ± SEM, 2-way ANOVA.

## Supplemental Table S4: Echocardiographic measurements of cardiac function in rSectm1a- and control IgG2a-treated mice at 1-month after cardiac I/R

| Parameter | Units | IgG2a-I/R (n = 6) | rSectm1a-I/R (n = 6) |
|---|---|---|---|
| Heart Rate (HR) | bpm | 550 ± 31 | 561 ± 23 |
| Diameter; s | mm | 2.55 ± 0.19 | 2.36 ± 0.12 |
| Diameter; d | mm | 3.54 ± 0.21 | 3.72 ± 0.13 |
| Volume; s | µL | 25.07 ± 4.68 | 20.16 ± 2.52 |
| Volume; d | µL | 54.58 ± 7.71 | 59.96 ± 4.91 |
| Ejection Fraction (EF) | % | 56.04 ± 2.36 | 65.98 ± 2.01* |
| Fractional Shortening (FS) | % | 27.97 ± 1.42 | 36.56 ± 1.75* |

*, $p < 0.05$, when comparing rSectm1a-I/R to IgG2a-I/R. Data are presented as Mean ± SEM, Student's *t* test.

## Supplemental Figures S1-12

### Figure S1A-C

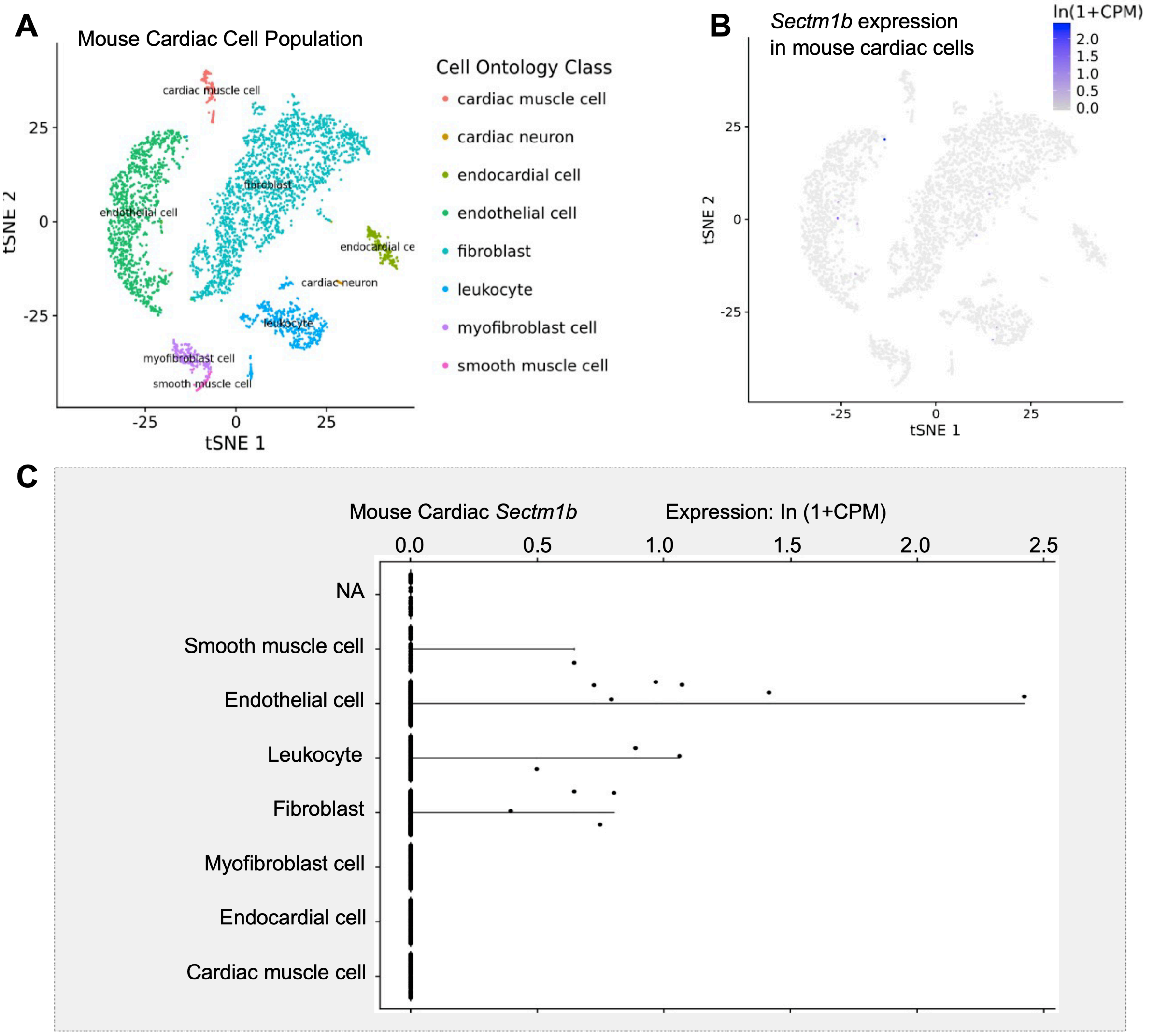


**Supplemental Fig.S1:** *Sectm1b* expression level in mouse cardiac cells. (**A-B**) Single-cell RNA-seq data from the Tabula Muris project (Reference #35) with t-distributed stochastic neighbor embedding (tSNE) plot of all cells isolated from murine hearts by FACS, (**C**) show that *Sectm1b* is highly enriched in cardiac endothelial cells, related to other cardiac cell types.

**Figure S2A-C**

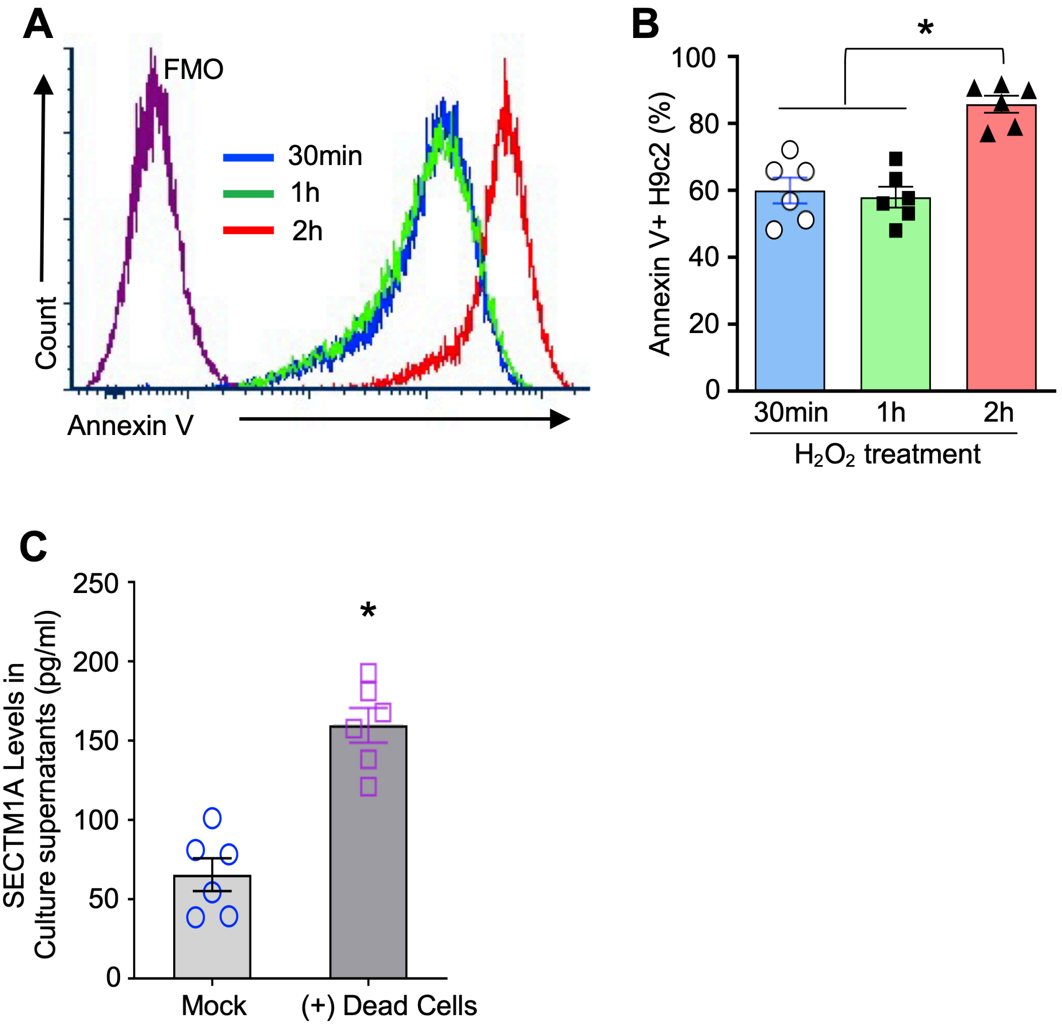


**Supplemental Fig.S2:** (**A/B**) Flow cytometry analysis with Annexin V staining. Representative flow cytometry histograms (A) and their quantification results (B) showing the apoptosis of H9c2 cells that is induced by $H_2O_2$ treatment for the indicated time points (n=6, *$p < 0.05$). (**C**) The concentration of SECTM1A was measured in culture supernatants collected from cultured BMDMs in the absence (mock control) and the presence of dead cells for 6h (n=6, *$p < 0.05$). All results are presented as mean ± SEM and analyzed by one-way ANOVA (A/B) or Student's *t* test (C).

**Figure S3A-G**

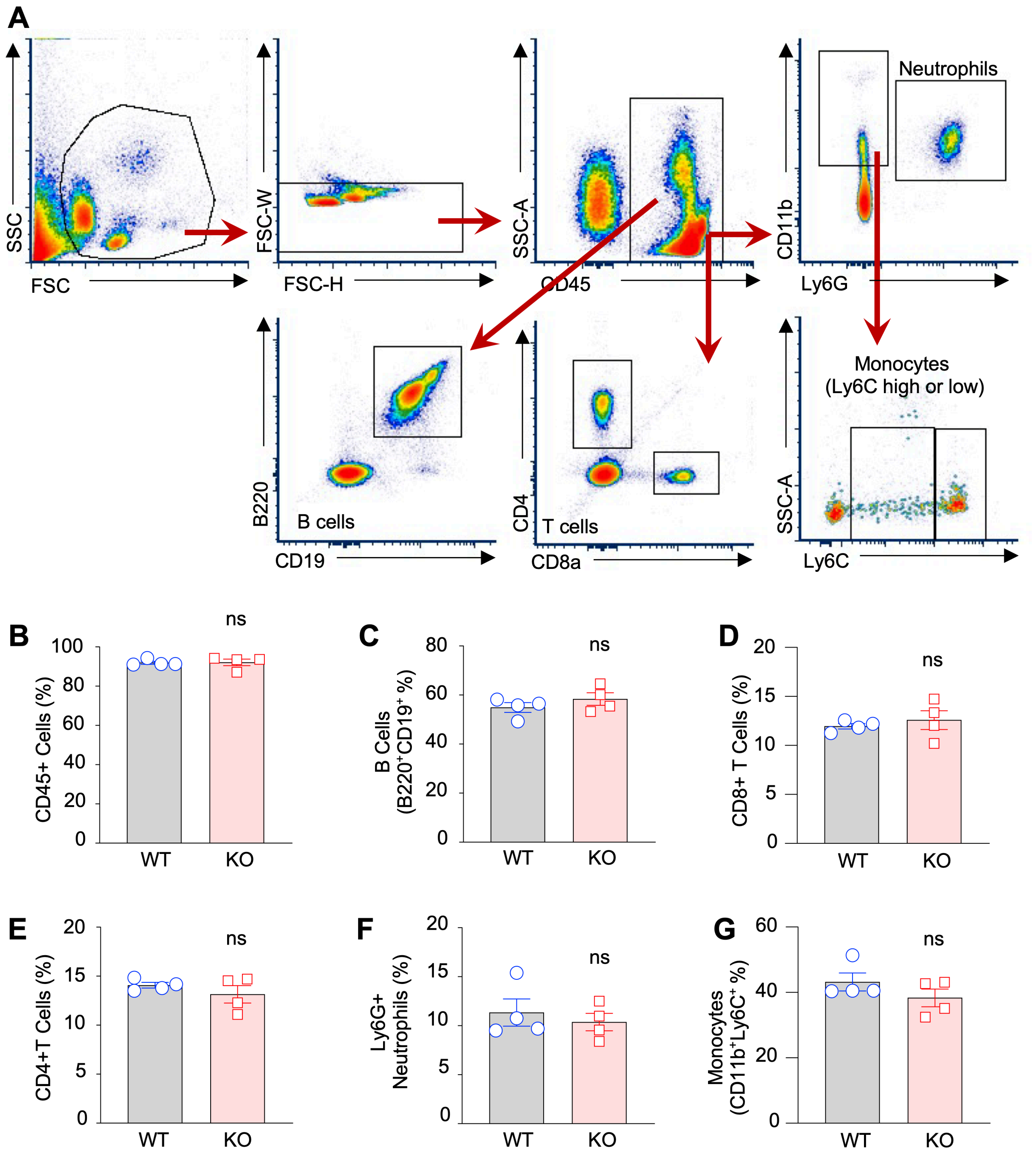


**Supplemental Fig.S3:** Analysis of immune cell populations in the blood of WT$^{Cherry}$ and KO$^{Cherry}$ mice. (A) Representative flow cytometry plots showing the gating strategy for different immune cell populations in murine peripheral blood. (B-G) Quantifications of different immune cell populations which show no significantly difference between two groups (n=4; ns, not significant *vs.* WT). Results are presented as mean ± SEM and analyzed by Student's *t* test (B-G).

**Figure S4A-H**

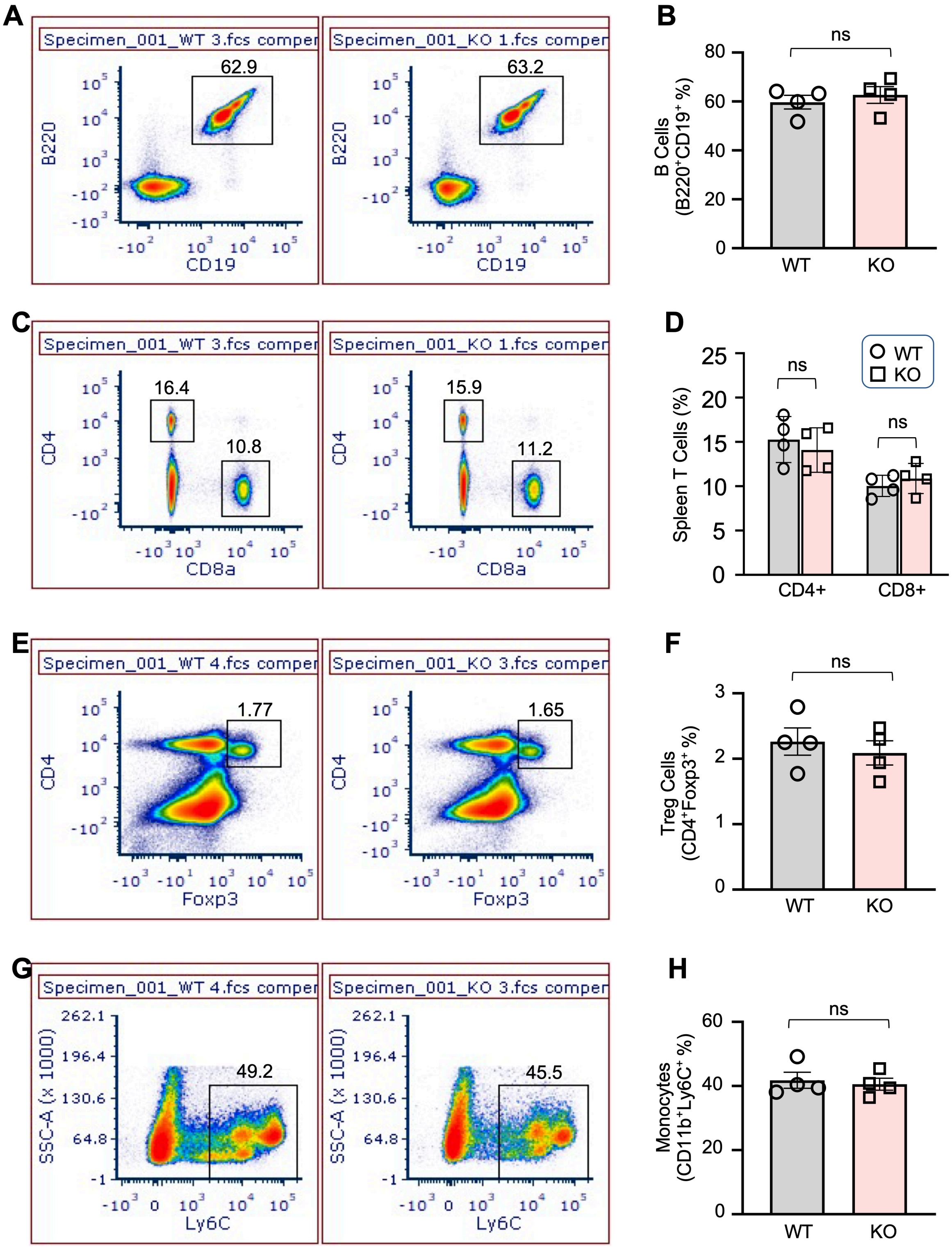


**Supplemental Fig.S4:** Analysis of immune cell populations in the spleen of $WT^{Cherry}$ and $KO^{Cherry}$ mice. (A, C, E, G) Representative flow cytometry plots and (B, D, F, G) their quantification results which show no significantly difference between two groups (n=4; ns, not significant.). Results are presented as mean ± SEM and analyzed by Student's *t* test (B, D, F, and H).

**Figure S5A/B**

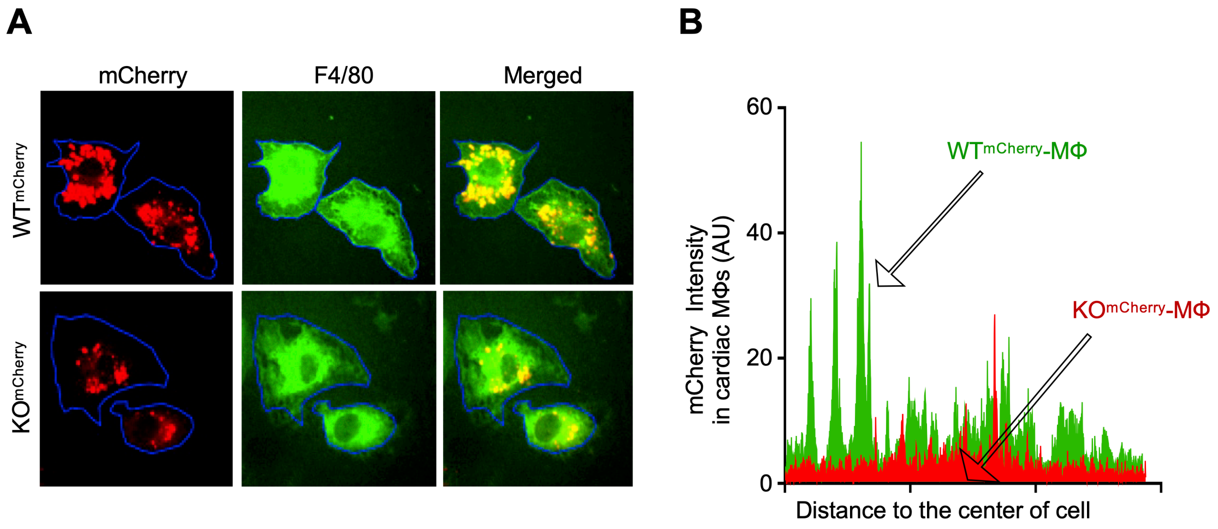


**Supplemental Fig.S5:** Cardiac MΦs isolated from $WT^{Cherry}$ and $KO^{Cherry}$ mice at Day 4 post-I/R and measured red mCherry intensity under confocal microscopy. (A) Representative images of mCherry-engulfing MΦs and (B) their quantification results.

**Figure S6**

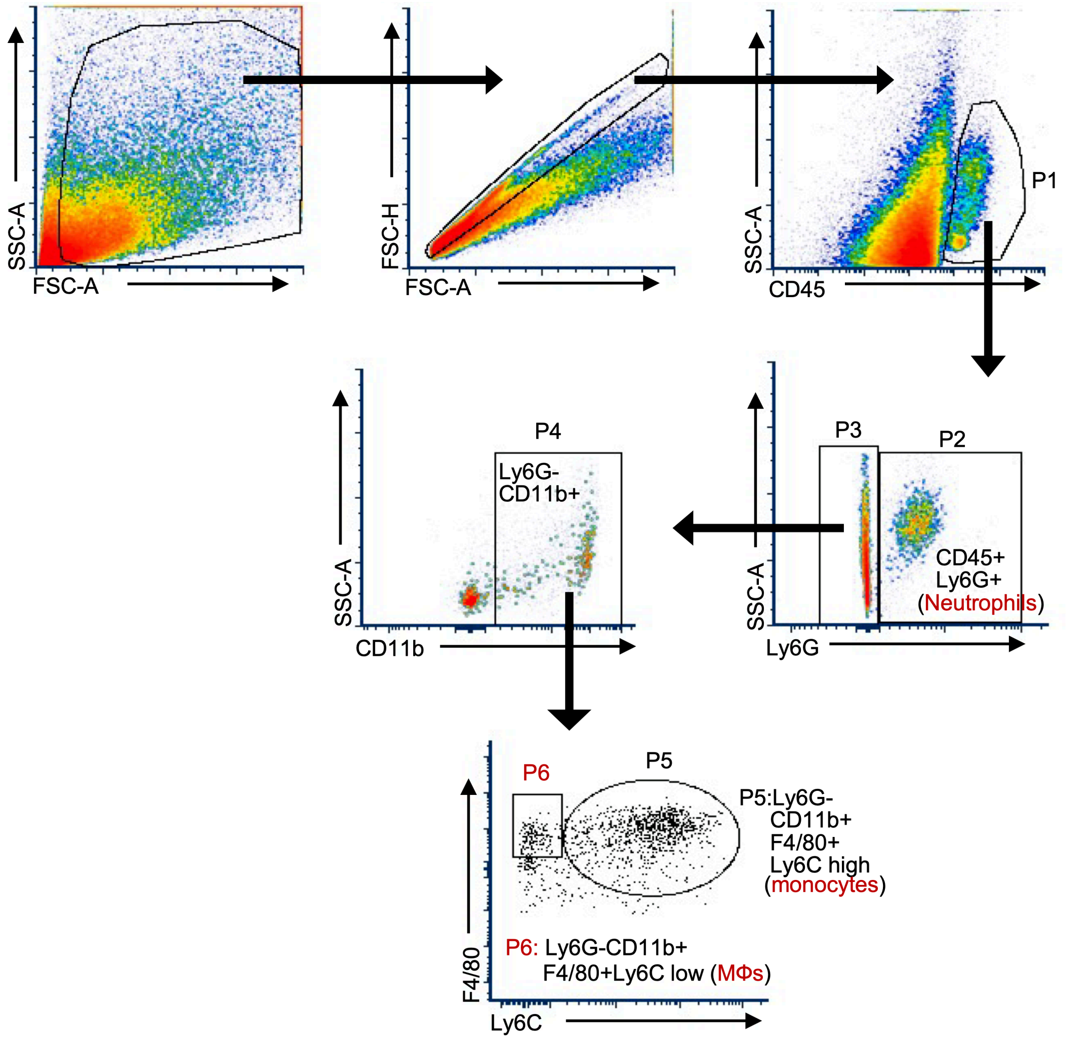


**Supplemental Fig.S6:** Representative flow cytometry plots showing the gating strategy for different types of immune cells isolated from murine hearts.

**Figure S7A-E**

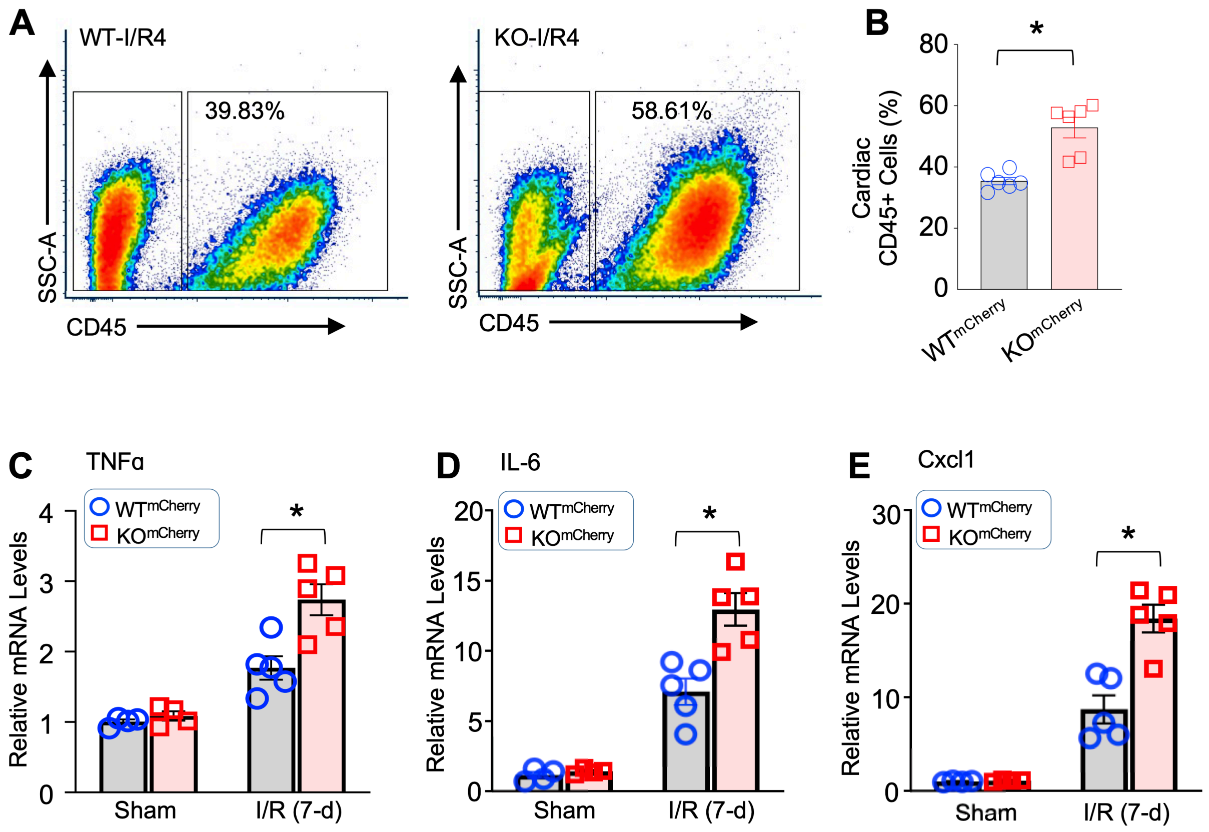


**Supplemental Fig.S7:** (**A**) Representative flow cytometry plots and (**B**) their quantification results showing higher number of CD45+ cells in KO$^{Cherry}$-hearts than WT$^{Cherry}$-hearts at Day 4 post-I/R (n=6; *, $p < 0.05$ *vs.* WT$^{mCherry}$). (C-E) The expression levels of pro-inflammatory cytokines (**C**) TNFα and (**D**) IL-6 as well as (**E**)chemokine Cxcl1 were measured in murine hearts after sham-operated and 7-day myocardial I/R (n=4 for sham group, n=5 for I/R group; *, $p < 0.05$ *vs.* I/R-WT$^{mCherry}$). Results are presented as mean ± SEM and analyzed by Student's *t* test (B-E).

**Figure S8A/B**

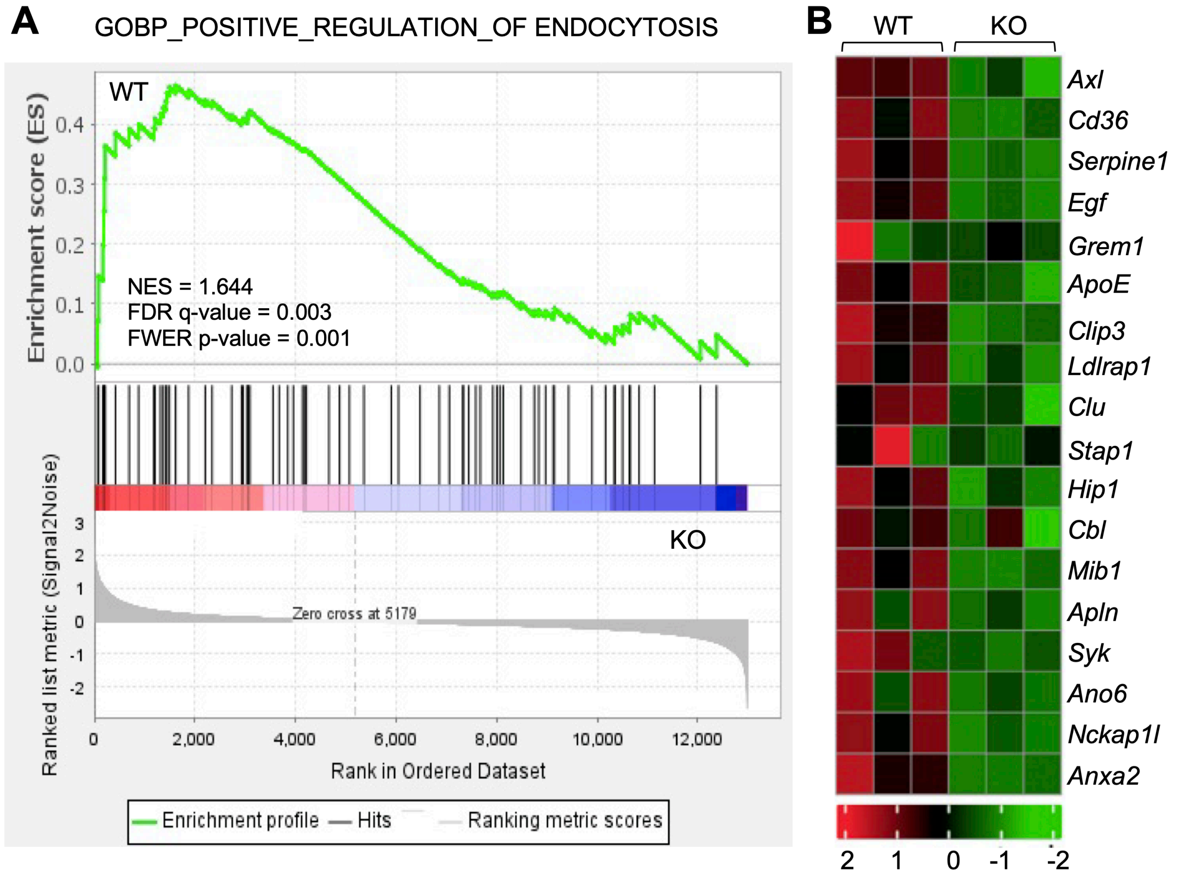


**Supplemental Fig.S8:** RNA-sequencing data assay. (A) Gene set enrichment analysis (GSEA) plot and (B) heatmap of positively regulated endocytosis genes in wild type (WT) MΦs compared to *Sectm1a*-knockout (KO) MΦs. Each square of the heatmap represents one biological replicate. Green and red color indicate down- *vs*. up-regulated genes, respectively.

**Figure S9A/B**

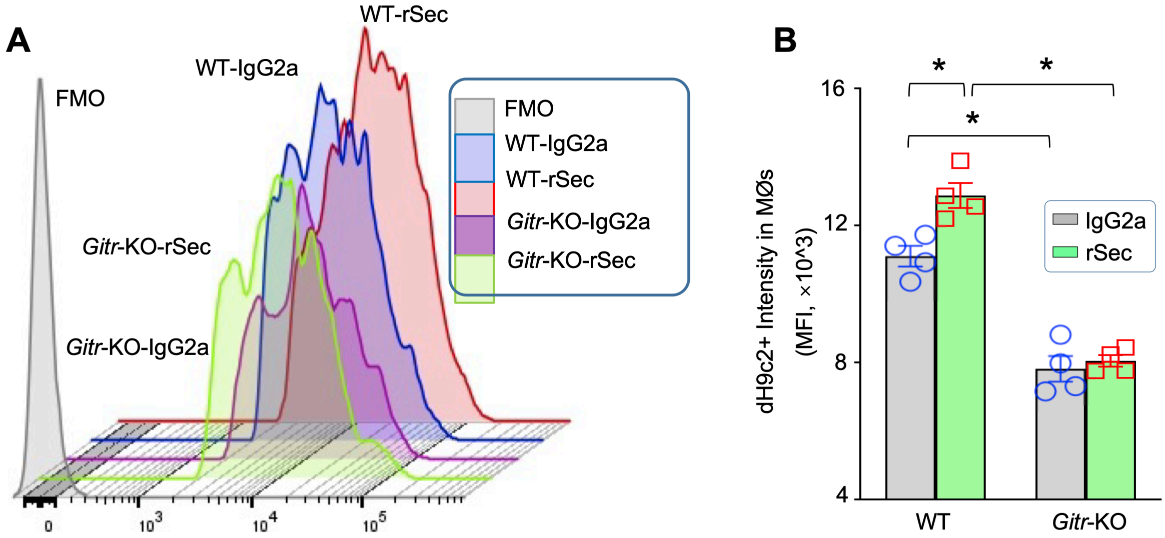


**Supplemental Fig.S9:** (**A**) Representative flow cytometry histograms and (**B**) their quantification of dead/dying H9c2-APC (deep red dye+) intensity (MFI) in MΦs (n=4; *, $p < 0.05$). Results are presented as mean ± SEM and analyzed by two-way ANOVA (B).

**Figure S10A-C**

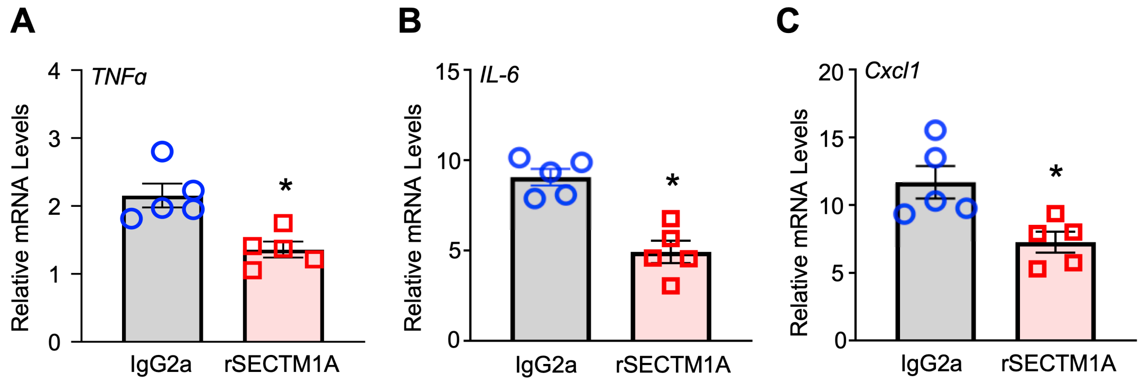


**Supplemental Fig.S10:** The expression levels of pro-inflammatory cytokines (A) *TNFα* and (B) *IL-6* as well as (C) chemokine *Cxcl1* were measured in IgG2a-treated and rSECTM1A protein-treated murine hearts after I/R (n=5; *, $p < 0.05$ *vs.* IgG2a-treated I/R hearts). Results are presented as mean ± SEM and analyzed by Student's *t* test (A-C).

**Figure S11A-F**

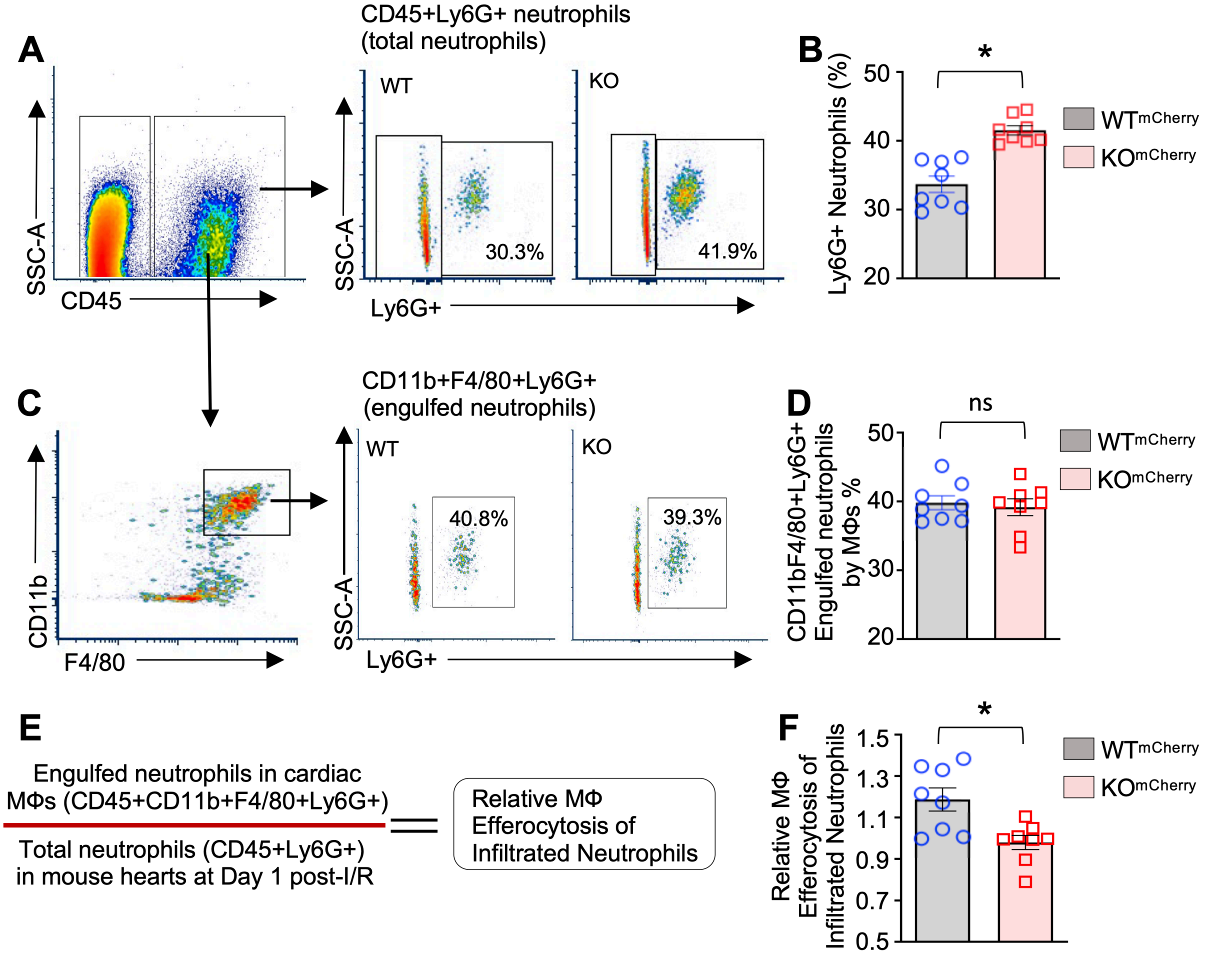


**Supplemental Fig. S11**: (A) Representative flow cytometry plots and (B) their quantification results showing higher number of Ly6G+neutrophils in KO$^{Cherry}$-hearts than WT$^{Cherry}$-hearts at Day 1 post-I/R. (C) Representative flow cytometry plots and (D) their quantification results showing the amount of cardiac MΦs in KO$^{Cherry}$-hearts than WT$^{Cherry}$-hearts at Day 1 post-I/R. (E/F) The ratio of engulfed to total neutrophils was calculated as relative MΦ efferocytosis of infiltrated neutrophils. (n=8; *, $p < 0.05$ *vs.* WT$^{mCherry}$). Results are presented as mean ± SEM and analyzed by Student's *t* test (B, D, and F). ns, not significant.

**Figure S12A-D**

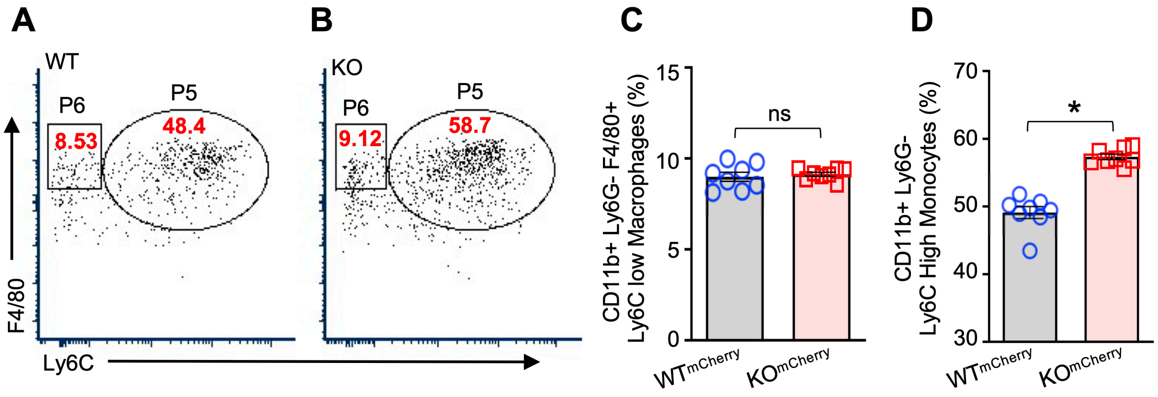


**Supplemental Fig. S12:** (A/B) Representative flow cytometry plots that used gating strategy of Supplemental S6, and (C/D) their quantification results showing higher number of monocytes, whereas similar number of MΦs in $KO^{Cherry}$-hearts, compared to $WT^{Cherry}$-hearts at Day 1 post-I/R. (n=8; *, $p < 0.05$ *vs.* $WT^{mCherry}$). Results are presented as mean ± SEM and analyzed by Student's *t* test (C-D). ns, not significant.
